# The Family Keeps on Growing: Four Novel Fungal OYEs Characterized

**DOI:** 10.3390/ijms23063050

**Published:** 2022-03-11

**Authors:** Marina Simona Robescu, Giovanni Loprete, Matteo Gasparotto, Filippo Vascon, Francesco Filippini, Laura Cendron, Elisabetta Bergantino

**Affiliations:** Synthetic Biology and Biotechnology Unit, Department of Biology, University of Padova, Viale G. Colombo 3, 35131 Padova, Italy; marinasimona.robescu@unipv.it (M.S.R.); giovanni.loprete@phd.unipd.it (G.L.); matteo.gasparotto.1@phd.unipd.it (M.G.); filippo.vascon@phd.unipd.it (F.V.); francesco.filippini@unipd.it (F.F.); laura.cendron@unipd.it (L.C.)

**Keywords:** old yellow enzyme, *Aspergillus niger*, *Botryotinia fuckeliana*, fungi, biocatalysis

## Abstract

Aiming at expanding the portfolio of Old Yellow Enzymes (OYEs), which have been systematically studied to be employed in the chemical and pharmaceutical industries as useful biocatalysts, we decided to explore the immense reservoir of filamentous fungi. We drew from the genome of the two Ascomycetes *Aspergillus niger* and *Botryotinia fuckeliana* four new members of the OYE superfamily belonging to the classical and thermophilic-like subfamilies. The two *Bf*OYEs show wider substrate spectra than the *An*OYE homologues, which appear as more specialized biocatalysts. According to their mesophilic origins, the new enzymes neither show high thermostability nor extreme pH optimums. The crystal structures of *Bf*OYE4 and *An*OYE8 have been determined, revealing the conserved features of the thermophilic-like subclass as well as unique properties, such as a peculiar N-terminal loop involved in dimer surface interactions. For the classical representatives *Bf*OYE1 and *An*OYE2, model structures were built and analyzed, showing surprisingly wide open access to the active site cavities due to a shorter β6-loop and a disordered capping subdomain.

## 1. Introduction

Old Yellow Enzymes (OYEs, EC 1.6.99.1) are a class of flavin-dependent ene-reductases (ERs) catalyzing the asymmetric hydrogenation of electronically activated C=C bonds in the presence of nicotinamide cofactors. As such, they are biocatalysts sought for effective synthesis methods designed and exploited in the pharmaceutical industry. They have been ubiquitously described and characterized at the genomic, structural, and catalytical levels in bacteria, animals, plants, and some yeast species [1]. In 2014, 60 genomes from the clades of Ascomycota and Basidiomycota were widely screened by Nizam et al. for the systematic research of OYE homologues, in which 424 putative OYE proteins were identified, and intriguingly, the number of OYE homologues found in each genome varied from 1 to 22 [2]. Such an evolutionary diversification was interpreted as a mechanism of fungal adaptation to different environments and carbon sources. The different isoenzymes may have evolved toward the recognition and reduction of specific substrates, as their expression is generally triggered by the surrounding environment (e.g., nutrients, presence of noxious substrates, or chemical-physical parameters) [2,3].

Filamentous fungi have recently attracted increasing attention as OYE reservoirs due to their unique features, and different whole-cell functional screenings have been reported. Carballeira et al. [4] analyzed the ability of 241 fungi to reduce the C=C bond of carvone. Out of the considered 241 fungi, just 3 of them (i.e., *Gongronella butleri*, *Schizosaccharomyces octosporus*, and *Diplogelasinospora grovesii*) were capable of reducing the model substrate with high productivity and stereoselectivity. Another functional screening was performed by Romagnolo et al. [3], evaluating the ability of 28 filamentous fungi belonging to different phyla (Ascomycota, Basidiomycota, and Zygomycota) to reduce 3 representative standard OYE substrates (cyclohex-2-en-1-one, α-methylnitrostyrene, and α-methylcinnamaldehyde). ER activity was widespread among all the screened organisms, as 27 fungi catalyzed the reduction of at least 1 of the 3 substrates tested. *Mucor circinelloides*, *Mucor plumbeus*, and *Gliomastix masseei* were the most versatile strains, showing the highest conversions for all the analyzed substrates. Avoiding an extensive review here, several other works showed the chemoselective reduction of α,β-unsaturated compounds using marine-derived fungi [5,6,7,8,9,10,11,12].

Fungi therefore appear to be valuable sources for ERs. Nevertheless, the number of isolated and characterized enzymes from these eukaryotic organisms is limited. These few examples are the OYE homologues FgaOX3 (or EasA) from *Aspergillus fumigatus* [13,14], FgaOX3Pc from *Penicillium* commune [15], and FgaOX3Pr3 from *Penicillium roqueforti* [16], which were produced as recombinant proteins, and their activity as ene-reductases was demonstrated in vitro in the reduction of chanoclavine-I aldehyde, an intermediate of the ergot alkaloid biosynthesis. Other studies have demonstrated the potential of fungal ERs to catalyze critical steps in the production of active pharmaceutical ingredients. Two examples are the synthesis of (*R*)-flurbiprofen, achieved at the semi-preparative scale by KYE2 from *Kluyveromyces marxianus* CBS4857 [17], and the asymmetric reduction of β-activated vinylphosphonates (fosfomycin and fosmidomycin analogues), carried out at the preparative scale by OYE3 from *Saccharomyces cerevisiae* [18].

Apart from the few examples reported in the literature, fungi are still a source of OYEs that is mostly unexplored. We have identified and produced four new putative ERs from the filamentous fungi *Aspergillus niger* CBS 513.88 and *Botryotinia fuckeliana* B05.10. We have characterized their substrate scope and biochemical properties, and obtained the crystal structure for one of them (a second crystal structure, the one for *Bf*OYE4, was anticipated in a submitted article by our group) [19]. This study thus broadens the present landscape of ene-reductases.

## 2. Results and Discussion

### 2.1. Identification of the New Putative ERs and Sequence Analysis

The Ascomycetes *Aspergillus niger* and *Botryotinia fuckeliana* were identified as promising sources for new putative ene-reductases by the genome-wide bioinformatic screening of Nizam et al. [2] and by the functional activity screening of Romagnolo et al. [3]. In the genomes of these fungi, different OYE homologues (i.e., 12 and 4) are present, and they belong to different OYE classes (Table S1). Furthermore, *A. niger* cells were shown to moderately reduce cyclohex-2-en-1-one and completely convert α-methyl-β-nitrostyrene within 2 days, while the *B. fuckeliana* cells were able to completely reduce cyclohex-2-en-1-one, and moderate activity was detected with α-methylcinnamaldehyde [3].

*An*OYE2 (XM_001393007) and *An*OYE8 (NT_166519) were chosen among the 12 OYE homologues present in the *A. niger* genome, being 42% and 30% identical to the OYE1 probe, respectively. Furthermore, *Bf*OYE1 (XP_001558622) and *Bf*OYE4 (XP_001554780) were chosen among the 4 OYE homologues present in the *B. fuckeliana* genome, as *Bf*OYE1 shows 43% identity and *Bf*OYE4 shows 27% identity with the OYE1 sequence.

**Figure 1:**
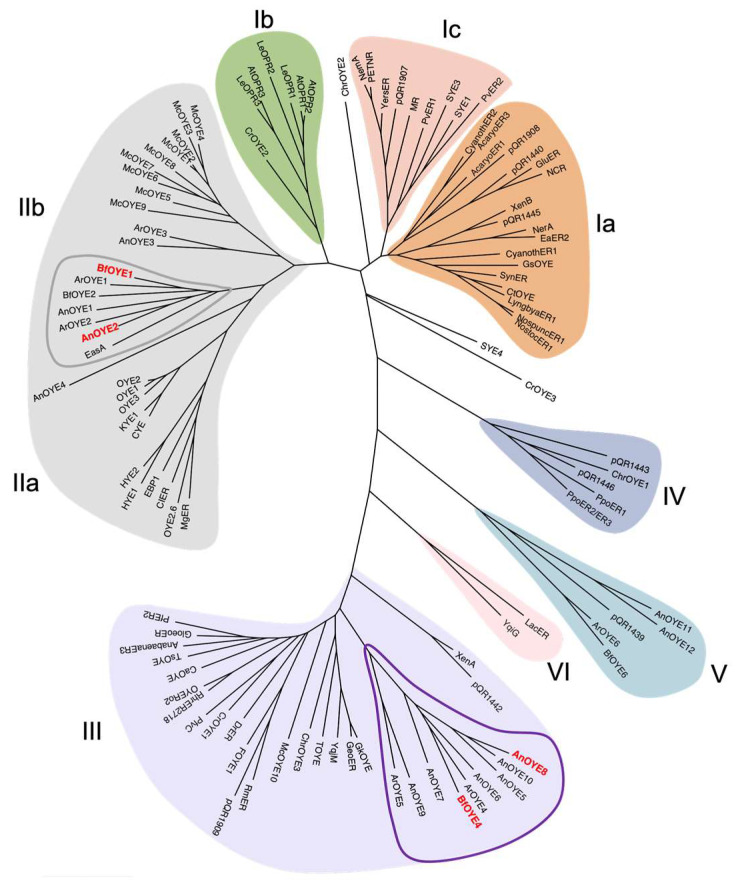
Phylogenetic analysis of fungal and previously described Old Yellow Enzymes. The evolutionary history conducted in MEGA11 was inferred by using the maximum likelihood method based on the JTT matrix-based model. The unrooted tree with the highest log likelihood (−68,148.01) is shown. Accession codes of all sequences used are reported in Table S2.

Based on the most recent classification [20], *An*OYE2 and *Bf*OYE1 belong to Class II OYEs, as they share all the fingerprint motifs described for “classical” OYE homologues identified in fungi. The alignments in Figure S1 show that both enzymes have the conserved residues involved in the FMN binding site architecture reported for OYE1 (P36, T38, G73, Q115, R244, G325, N326, F327, G346, G348, and R349) [21]. Notably, up to the present update, Class II is exclusively populated by members of the Ascomycota phylum of fungi (Figure 1).

On the other hand, *An*OYE8 and *Bf*OYE4 share most of the typical conserved residues reported in Class III OYEs (S23, P24, C26, A60, Q102, R215, S249, Q265, G284, M285, F305, G307, R308, and R312; YqjM numbering) (Figure S1). However, some differences can be found in the C-terminal sequence involved in monomer-monomer interaction and dimer formation. The arginine finger (R336 in YqjM) that protrudes into the active site of each monomer and contributes to defining the key features of the catalytic pocket is substituted, both in *An*OYE8 and *Bf*OYE4, by a bulkier hydrophobic amino acid such as tryptophan, as also seen in Ppo-ER3 (W376) (Figure S1) and XenA (W358). Generally, Class III OYEs isolated from extremophile bacteria have shorter amino acid sequences (337–371 residues) than Class I or Class II homologues (349–412) and a higher content of proline residues in their loops and turns [22]. *An*OYE8 and *Bf*OYE4 are 439 and 421 amino acids long, respectively, and they have a lower proline content (<7%) if compared with thermophilic Class III homologues, especially the one isolated from the *Thermus* species. The longer N-terminal loops (blue stretch in Figure S1) can be a common feature of fungal enzymes (i.e., putative signal peptides targeting the enzyme to the appropriate subcellular compartment). Nizam et al. [2] reported a mitochondrial localization for *Bf*OYE4 protein and a cytoplasmatic one for *An*OYE8. Longer sequences, longer N-termina, and a low proline content (see forward, Table S3) are peculiar characteristics that make the new members cluster in an isolated fungal (Ascomycota) sub-clade of Class III, which exclusively groups OYEs of a bacterial origin (Figure 1).

Based on the genome-wide screening performed by Nizam et al. [2] on a great variety of OYE sequences present in fungi, a third class of OYE homologues was identified (Class V in Figure 1) with peculiar FMN interactions and active site organization [21]. Two OYE homologues from *A. niger* (*An*OYE11 and *An*OYE12) and one homologue from *B. fuckeliana* (*Bf*OYE6) belong to this new class (see Table S1). However, to our knowledge, this class of OYEs is still unexplored.

### 2.2. Production and Purification of the Recombinant Proteins

All the proteins were produced in the heterologous host *E. coli* BL21 (DE3) with an N-terminal His_6_-tag. *Bf*OYE1 was the only protein to be handily purified with very good yields (40 mg/L) and homogeneity (Figure S2) when 25 µM of riboflavin was added to the cell culture media. In the absence of this FMN precursor, the protein was obtained in lower amounts (Figure S3). The addition of the cofactor precursor was also employed for the production of the other OYE homologues. However, both lowering the growth temperature after induction and trying different *E. coli* strains endowed with bacterial chaperons were necessary to increase the yield (*Bf*OYE4, as already reported in Robescu et al. [19]) or improve solubility (*An*OYE2 and *An*OYE8).

Initially, the expression of the *An*OYE2 gene was attempted in BL21 (DE3) cells at 25 °C and 16 °C. The protein was highly over-expressed but completely insoluble at 25 °C, and only a small fraction was soluble at 16 °C (Figure S4, panel A). The *E. coli* Arctic^®^ strain resulted in being the most convenient, with a high amount of soluble protein obtained at 16 °C (Figure S4, panel B). *An*OYE8 was synthesized at similarly high levels in BL21 (DE3) cells at 25 °C, but its solubility was very low (Figure S5, panel A). In the *E. coli* BL21 (DE3) Arctic^®^ strain grown at 12 °C, its solubility was clearly enhanced, but a major insoluble fraction was still present (Figure S5, panel B). Both proteins were finally purified at a low temperature from the *E. coli* BL21 (DE3) Arctic^®^ cells in moderate yields (13 mg/L *An*OYE2 and 9 mg/L *An*OYE8). The chaperonin Cpn60 co-expressed by the Arctic^®^ strain was co-purified as a major contaminant of *An*OYE2 and *An*OYE8. Even though most of the undesired contaminant was removed (Figures S4 and S5, panel C, lane Chp) following a standard protocol [23], some contaminant chaperonin molecules remained in the final preparation. With the goal of favoring the folding of these proteins in vivo and detaching them from the undesired Cpn60, for further crystallization trials, we increased the concentration of riboflavin added to the culture media up to 100 μM. With this strategy, the solubilities of both proteins in the *E. coli* BL21 (DE3) cells were enhanced, with yields of 18 mg/L for *An*OYE2 and 10 mg/L for *An*OYE8 (Figure S2, panel A and Figure S2, panel B, respectively).

The UV–visible absorbance spectra of the purified proteins displayed a shift in the maximum peak absorbance upon thermal denaturation. After boiling the samples for 15 min at 90 °C, the supernatant turned bright yellow, and the maximum of its absorbance spectrum shifted to 446 nm (Figure S6), corresponding to the maximum of the released free FMN.

### 2.3. Oligomeric State Determination

**Figure 2:**
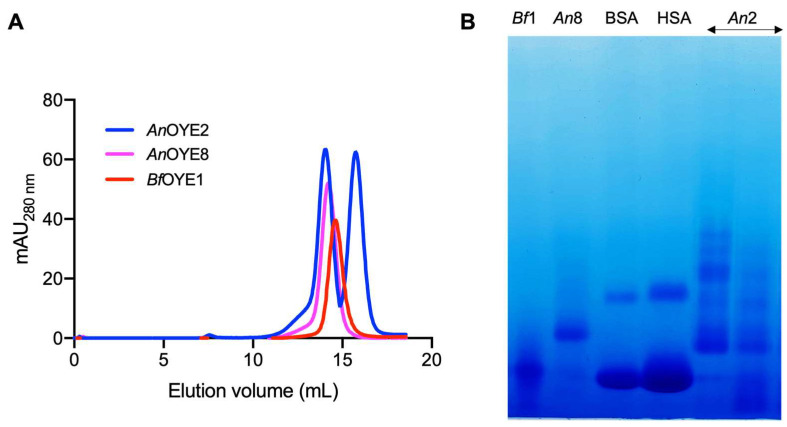
Analytical gel filtration of purified protein samples (150 µg) eluted from a Superdex 200 10/300 GL column (**A**) and blue native gel (BN-PAGE) of samples (12.5 µg) from each observed peak (**B**). BSA = bovine serum albumin (25 µg); HSA = human serum albumin (30 µg).

Following examination of the size and purity by SDS-PAGE, the oligomeric states of the recombinant *An*OYE2, *An*OYE8, and *Bf*OYE1 proteins were investigated by analytical gel filtration chromatography (Figure 2A; see calibration curve in Figure S7). *Bf*OYE1 (43.5 kDa) was eluted as a single peak in solution with an apparent molecular weight of 57 kDa, corresponding to a retained dimer (as confirmed by BN-PAGE, Figure 2B). *An*OYE2 (43.3 kDa) showed the presence of both retained monomeric and dimeric or oligomeric species (as suggested by BN-PAGE, Figure 2B).

For the Class III *An*OYE8 enzyme (47.9 kDa), analytical gel filtration analysis revealed a single peak with an apparent molecular weight of roughly 1.3-fold the monomer, once again in between the dimeric and monomeric expected retention times. This result can be interpreted again as the retention by the matrix of dimeric fractions (as observed in BN-PAGE, too; Figure 2B). Indeed, in the crystal structure determined in this work, *An*OYE8 formed a dimeric species whose architecture agreed with the typical quaternary organization of Class III OYEs.

For *Bf*OYE4, a heterogeneous profile in size exclusion chromatography suggesting the presence of different oligomeric species in solution was already reported [19]. Analogous to *An*OYE8, in the crystal structure of *Bf*OYE4, the typical dimeric architecture could be observed [19].

Generally speaking, members of the Class I and Class II OYEs (“classical homologues”) were found to occur in solution exclusively as monomers or homodimers. On the other hand, class III OYEs (“thermophilic-like homologues”) usually occurred as homodimers or homotetramers in solution. Higher species (octamers and dodecamers) were observed in some thermophilic homologues (e.g., TOYE [24] and *Ts*OYE [25]), and monomeric species were found in non-thermostable homologues as well (e.g., *Rm*ER [26] and *Ca*OYE [27]) (Table S3). It has been reported that the equilibrium between different oligomeric species in OYEs depends on the protein concentration as well as the flavin redox state [28].

### 2.4. Substrate Spectra

To explore the biocatalytic potential of the newly identified ene-reductases, different standard substrates were tested spectrophotometrically by measuring the consumption of NADPH at 340 nm. The standard substrates belong to different types of molecular classes activated by different electron-withdrawing groups (e.g., linear and cyclic ketones, as well as aldehydes and maleimides). The results from such an evaluation are summarized in Table 1.

The non-substituted five-member ring cyclopent-2-en-1-one (**1**) was reduced with lower reaction rates by *Bf*OYE enzymes compared with the non-substituted six-member ring cyclohex-2-en-1-one (**2**) (3.3–10.3 U/mg vs. 2.2–3.8 U/mg). The same enzymes converted the α-substituted 2-methyl-cyclohex-2-en-1-one (**3**), with *Bf*OYE1 showing fourfold higher activity (5.7 vs. 1.4 U/mg) than *Bf*OYE4. The reduction of ketoisophorone (**4**) was detected just in the presence of *Bf*OYE1 with a rate comparable to those of the other accepted substrates (10.3 U/mg). *Bf*OYE1, *Bf*OYE4, and *An*OYE2 demonstrated themselves to be active in the presence of maleimide (**5**) (11.3, 22.5, and 16.6 U/mg, respectively). Curiously, *An*OYE8 appeared to be totally inactive on this substrate, which is generally considered a typical substrate of ene-reductases. The activity of all four enzymes with **5** was also tested in the presence of NADH, but a maleimide reduction was undetectable, suggesting a strong preference for the NADPH cofactor. Three linear substrates were also taken in the exam: (*E*)-2-methylpent-2-enal (**6**), (*E*)-hex-2-enal (**7**), and oct-1-en-3-one (**8**). Among all measures, significant activities were registered for *Bf*OYE1, with *Bf*OYE4 showing a singularly high activity only with **8**.

Overall, the enzymes isolated from *B. fuckeliana* had a wider substrate spectrum and higher specific activities compared with the ones isolated from *A. niger*. Even if the *A. niger* cells were shown to be active in the reduction of cyclohex-2-en-1-one (**2**) and (*Z*)-β-methyl-nitrostyrene [3], in our hands, *An*OYE2 and *An*OYE8 seemed to have a restricted substrate spectrum. Particularly, the former enzymes appeared to be the most selective one among those studied here. Since the genome of this Ascomycete encodes 12 putative OYEs, high substrate specialization for each isoenzyme and expression triggered by different substrates or conditions can be conceivably envisaged. Indeed, such a hypothesis has already been put forward for two other fungal species: *Ascochyta rabiei* (Ascomycete) and *Mucor circinelloides* (Mucoromycete). In both cases, genome analysis found 6 [21] and 10 [29] putative OYEs, respectively, for which the modeling-predicted structural differences suggested specific substrate-enzyme matching. Afterward, the expression profiles of the genes encoding the putative isoenzymes were analyzed for both species and clearly showed differential activation in response to environmental stimuli (plant infection or oxidative stress for *A. rabiei*) [2,21] or exposure to different substrates (for *M. circinelloides*) [30]. Thus, the low activities registered for our two *An*OYEs could be ascribed to the fact that standard substrates generally used during screenings of OYEs may not mimic the natural substrates (still unknown) of these enzymes, leading to a limited activity spectrum and low specific activities being recorded.

### 2.5. Steady State Kinetic Parameters

Given the substrate preferences and specific activities observed during the preliminary screening, the kinetic constants were determined for the best substrates (Table 2). The enzymatic properties of three out of the four new OYEs were rapidly measured by spectrophotometric assays. This could not be managed with *An*OYE8 due to the very modest activity registered for all the substrates tested.

For *An*OYE2, the kinetic constants were determined, with just the preferred substrate maleimide (**5**) showing good catalytic efficiency (738 mM^−1^ s^−1^). In addition, with maleimide, the highest affinities (K_M_ 0.01 mM and 0.07 mM) and activities (k_cat_ = 9.91 s^−1^ and 36.35 s^−1^) were registered for both *Bf*OYE1 and *Bf*OYE4, respectively, resulting in high catalytic efficiencies. Cyclohex-2-en-1-one (**2**) was reduced by both the *Bf* enzymes with catalytic efficiencies in the range of 3.9–11.4 mM^−1^ s^−1^. The introduction of the methyl group in the α position (**3**) of cyclohex-2-en-1-one reduced their activity toward the substrate, as already found in the preliminary screening. In particular, *Bf*OYE1 could better tolerate the methyl substitution on the ring than *Bf*OYE4, with a catalytic efficiency of 11.4 vs. 3.9 mM^−1^ s^−1^. Other bulkier substitutions, such as that in ketoisophorone (**4**), reduced the catalytic efficiency almost fourfold compared with **2** for *Bf*OYE1 and zero for *Bf*OYE4. The steric hindrances appeared to be significant for the activity on the aromatic compounds. Indeed, the ring size also had a meaningful effect on the kinetic parameters, with the catalytic efficiencies observed for both enzymes with cyclopent-2-en-1-one (**1**) being very low (1.0–2.1 mM^−1^ s^−1^) compared with the ones for cyclohex-2-en-1-one (**2**). *Bf*OYE1 accepted as substrates the aliphatic enals (*E*)-2-methylpent-2-enal (**6**) and (*E*)-hex-2-enal (**7**) (112.0 and 23.6 mM^−1^ s^−1^, respectively) but not the aliphatic enone oct-1-en-3-one (**8**). The latter compound was instead transformed with discrete efficiency by *Bf*OYE4 (8.3 mM^−1^ s^−1^). Finally, for *An*OYE2, *Bf*OYE1, and *Bf*OYE4 the kinetic parameters for the preferred cofactor NADPH were also determined. *An*OYE2 and *Bf*OYE1 showed very good catalytic efficiencies (216.2 mM^−1^ s^−1^ and 233.2 mM^−1^ s^−1^, respectively). *Bf*OYE4 showed a lower affinity (K_M_ = 0.13 mM) but high activity (k_cat_ = 89 s^−1^) for NADPH.

### 2.6. pH Optimum and Thermal Stability

Other relevant parameters such as the pH optimum and melting temperature were investigated.

*Bf*OYE1 and *An*OYE8 showed their maximum activity in a limited pH range of between 6.0 and 7.0 and between 7.0 and 8.0, respectively, compared with *Bf*OYE4 [19] and *An*OYE2 (100% activity from pH 6.0 to pH 10.0) (Figure S8). While *Bf*OYE1 was still active at extremely acidic pH values (e.g., 35% of activity was retained at pH 4.0), *An*OYE8 seemed sensitive to acidic and basic pH values.

The stability of all proteins was evaluated by detecting the apparent melting temperature (T_m_) using the Thermofluor method in the presence of various co-solvents: ethanol, acetone, acetonitrile, dimethyl sulfoxide, and dioxane. As reported in Figure S9, *An*OYE2 and *Bf*OYE1 showed T_m_ values in aqueous buffer at pH 8.0 of 41.5 °C and 42 °C, respectively. Both enzymes could tolerate low percentages of organic co-solvents (5% *v*/*v*), even if *An*OYE2 showed good stability in up to 40% *v*/*v* dimethyl sulfoxide. A decrease in stability was observed in high percentages (10–40% *v*/*v*) for the other co-solvents. Although clustering in the thermophilic-like Class III, *An*OYE8, and *Bf*OYE4 showed T_m_ values of 44 °C and 43 °C, respectively, similar to other enzymes categorized into the same subclass but isolated from mesophilic organisms and reported in the literature as non-thermostable (Table S3). *An*OYE8 was very stable up to 50% *v*/*v* dimethyl sulfoxide, but a decrease in its stability was measured in all the other co-solvents even in low percentages. Conversely, *Bf*OYE4 could tolerate up to 30% *v*/*v* of acetone, acetonitrile, and dimethyl sulfoxide well.

### 2.7. AnOYE8 X-ray Crystal Structure

**Figure 3:**
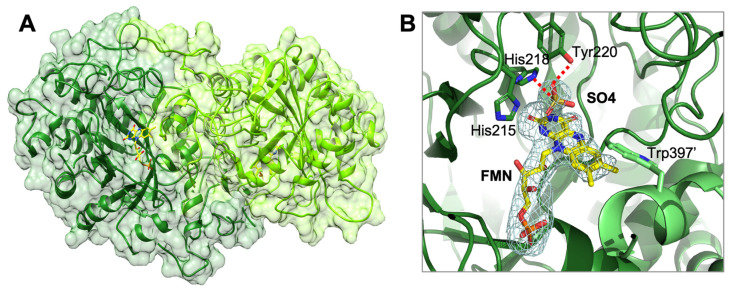
Cartoon representation of the crystal structure of *An*OYE8 (PDB: 7QFX): (**A**) surface representation showing the two protein chains forming the dimeric architecture; (**B**) details of *An*OYE8 active site, where the ligands omit map (FMN and SO_4_) was calculated by Phenix and is shown contoured at 2.5 σ. The two protomers of *An*OYE8 are colored in forest green and light green. FMN cofactor and sulphate anion (SO_4_) bound in the active site are shown with C atoms in yellow and orange, respectively. Most relevant residues of the catalytic cavity are shown with forest green C atoms, while Trp397′ of the adjacent protomer is shown with light green C atoms, red for O, and blue for N. Hydrogen bonds are shown as red dashed lines.

The crystal structure of *An*OYE8 (PDB 7QFX) was determined to a maximum resolution of 2.8 Å. *An*OYE8 was solved and refined in the C121 space group with four molecules per asymmetric unit (Table S4). The enzyme presented both the typical features of Class III OYE homologues and peculiar properties. The overall structure of a single subunit showed the typical conserved α8/β8-TIM barrel fold (Figure 3A). In agreement with the general behavior of Class III OYEs, the four molecules within the asymmetric unit assembled into dimers with the functional quaternary architecture peculiar of these enzymes. *An*OYE8 is an elongated dimer composed of two protomers (Figure 3A) with an interacting surface of 2807 Å^2^ (data obtained by EMBL-EBI-PISA analysis [31]). Both the α1-helix and loop 3 (capping subdomain), as well as the peculiar long N-terminal and C-terminal loops of each protomer, were involved in the dimerization surface, which contained 71 interfacing residues that established 18 hydrogen bonds and 8 salt bridges. The contribution to solvation free energy upon complex formation due to van der Waals contacts and hydrophobic interactions amounted to −30.9 kcal/mol (average values within the asymmetric unit). The C-terminus of each subunit (from Gly393 to Lys416) protruded through an “amino acid finger” (Trp397) that reached the active site of the adjacent subunit, shaping the entrance and the catalytic cavity of each subunit (Figure 3A). *An*OYE8 accommodated a non-covalently bound FMN cofactor molecule lying at the bottom of the active site pocket. As expected, the *si*-face of the FMN was exposed to the solvent while the *re*-face was in contact with the protein backbone. The cofactor was bound via extensive hydrogen bonding and hydrophobic interactions with side as well as main chain elements (Figure S10). Similar to other Class III OYEs, *An*OYE8 contained the highly conserved proton donor Tyr220 and the histidine pair (His215 and His218) involved in the binding and proper orientation of the substrate through hydrogen bonding with the electron-withdrawing group of the substrate (Figure 3B). The omit electronic density map revealed the presence of a small organic molecule, most likely SO_4_ anion, present within the crystallization precipitant agents, lying on top of the isoalloxazine ring of FMN (Figure 3B). In the complex with SO_4_, two oxygens of the compound formed two hydrogen bonds with the side chain of His215 and Tyr220, thus confirming the importance of these residues in substrate orientation and catalysis.

To our knowledge, a single crystal structure of Class III OYEs isolated from filamentous fungi has been reported so far: *Bf*OYE4, crystalized by our group and described elsewhere [19]. As previously shown in the phylogenetic tree (Figure 1), *An*OYE8 and *Bf*OYE4 were grouped along with other putative fungal OYEs into a sub-clade of Class III, distinct from other well-known bacterial enzymes. Indeed, the two structures show high structural similarity with an r.m.s.d. of 0.712 Å (Figure S11), and both are characterized by protein sequences longer than those of the other enzymes of the clade (421 and 439 amino acids, respectively), as underlined above. In both cases, the longer N-terminal and C-terminal loops contribute to forming the dimer interface and shaping the active site architecture, respectively (Figure S12). In particular, the C-terminus of both proteins protrudes through a Trp finger (and not an Arg finger, as reported for the majority of bacterial Class III homologues) into the adjacent protomer, partially reducing the accessibility of the active site pocket (this effect being more pronounced in *An*OYE8 than *Bf*OYE4). CASTp analysis confirmed that the volume of the active site cavity was slightly smaller in *An*OYE8 (861.24 Å^3^) compared with *Bf*OYE4 (1117.2 Å^3^), which indeed has a more exposed catalytic pocket (Figure S12).

**Figure 4:**
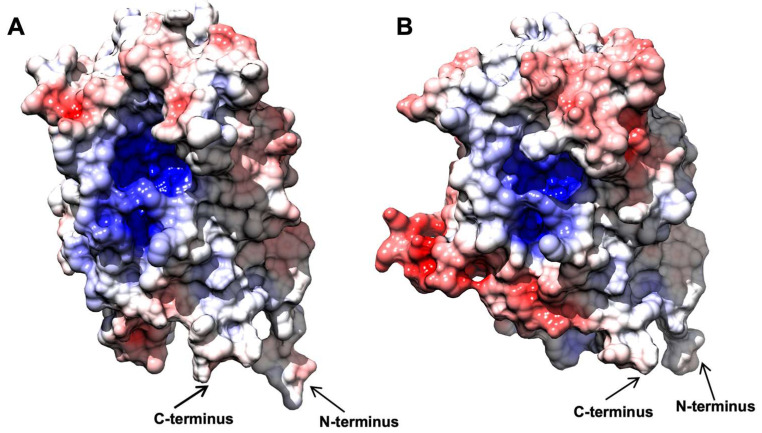
Comparative analysis of surface charge distribution, calculated at 150 mM NaCl, pH 8.0, and 310 K. *An*OYE8 is on the left (**A**), and *Bf*OYE4 is on the right (**B**). Density of negative potential is red, positive is blue, and neutral is white.

When the electrostatic surface potential was considered, the two structures appeared to be quite similar (Figure 4), as well as their theoretical isoelectric points (Table S1). In both cases, the inner surface of the barrel was found to be heavily positive, in agreement with its affinity to the negatively charged FMN molecule. The outer surface of both enzymes showed some differences, with *Bf*OYE4 presenting negatively charged spots more pronounced than those in *An*OYE8. Around the active site cavities, the surface seemed to be slightly positively charged in both proteins. Distinctive features in the charge distributions of *An*OYE8 and *Bf*OYE4 with respect to other members of ClassIII can be further appreciated in Figure S13.

### 2.8. AnOYE2 and BfOYE1 Model Structures

**Figure 5:**
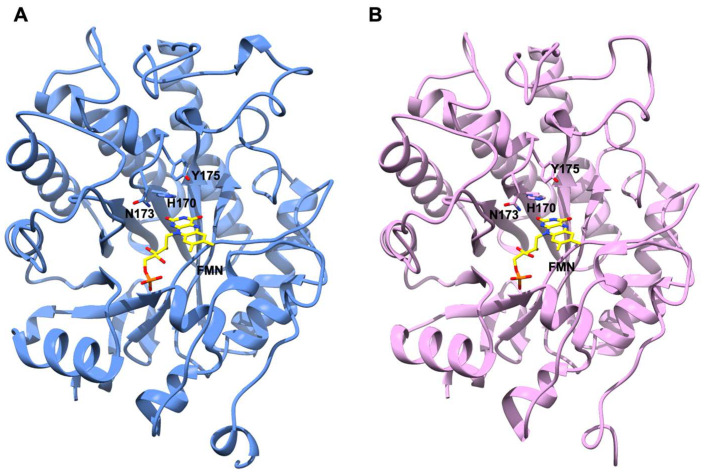
Model structures of *An*OYE2 (cornflower blue) (**A**) and *Bf*OYE1 (pink) (**B**). FMN cofactor docked in the active site is shown with C atoms in yellow. The conserved catalytic residues His170, Asn173, and Tyr175 are shown as sticks in both models.

Different crystallization conditions and experimental strategies were attempted in order to determine the 3D crystal structures of *An*OYE2 and *Bf*OYE1. Even if we managed to obtain crystals for both proteins, no diffraction was observed. To obtain some insights from these Class II homologues, we decided to model their structures. To this aim, we used the highly homologous EasA crystal structure (PDB 4QNW) as a template (50% and 52% sequence similarity, respectively). The values in Table S5 indicate an overall good quality of the models as, in both cases, the GMQE index (which indicates the Global Model Quality Estimation) was close to 1, and almost 95% of the residues displayed Ramachandran favored orientation. The high identity and coverage with the template allowed the modeling of highly homologous structures, as indicated by the low r.m.s.d. between the models and the template. Overall, the models shared a conserved architecture where eight α-helices surrounded a TIM barrel domain composed by eight twisted β-strands. Both proteins were endowed with an N-terminal β-hairpin loop which closed one side of the barrel and defined a cavity inside both molecules (Figure S14). CASTp analysis revealed that this cavity was slightly smaller in *Bf*OYE1 (506.49 Å^3^) than in *An*OYE2 (578.96 Å^3^). Docking simulations performed with COFACTOR showed that both pockets were able to fit an FMN cofactor molecule allocated as observed with other OYEs. Sequence alignment (Figure S1) and a Ligplot analysis of the homology models (Figures S15 and S16) showed that the residues involved in H-bonds, charge-charge, and van der Waals interactions were highly conserved. The cofactor molecule was roughly perpendicular to the barrel axis, with its *si*-face exposed to the solvent and the *re*-face buried inside the protein core (Figure 5). A comparison with the template structure EasA also highlighted that the orientation of the catalytically active residues His173, Asn176, and Tyr178 (EasA numbering) was conserved in both models, suggesting that both proteins can favorably catalyze a hydride transfer from N5 of the reduced flavin cofactor to a substrate with an activated C=C-bond (Figure 5). Interestingly, due to a shorter loop 6 (which could not protrude into the active site cavity until the FMN binding core) and a disordered loop 3 (capping subdomain, which also showed an open orientation compared with other Class II homologues), both proteins showed a highly exposed active site cavity that left wide access to the substrates and solvent (Figure S17). Such wide open access was measured by the pseudo-atom distance between the loop β2 “bounding” residues and the C-terminus extension [32] involved in shaping the bottom of the binding pocket (e.g., Tyr82 and Tyr375 in OYE1). This was significantly larger in *An*OYE2 and *Bf*OYE1 as well as in EasA (about 13–14 Å) than in other Class II enzymes (around 8–9 Å) (Figure S18).

**Figure 6:**
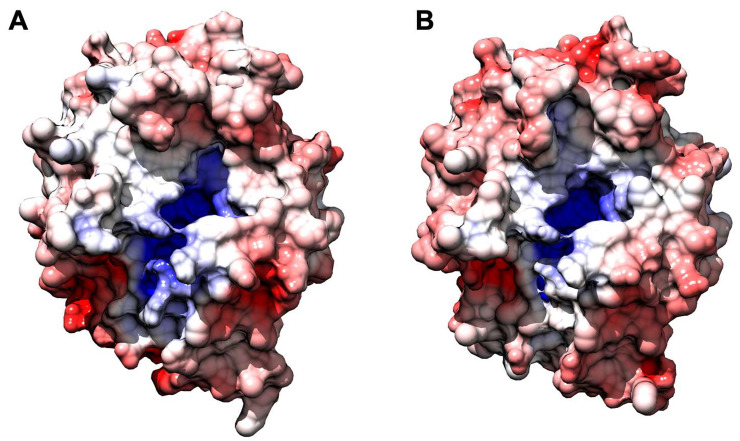
Comparative analysis of surface charge distribution calculated at 150 mM NaCl, pH 8.0, and 310 K. *An*OYE2 is on the left (**A**), and *Bf*OYE1 is on the right (**B**). Density of negative potential is red, positive is blue, and neutral is white.

*Bf*OYE1’s isoelectric point was slightly more acidic (5.48) than that of *An*OYE2 (6.02) (Table S1). However, both models showed a quite similar distribution of the electrostatic surface potential (Figure 6). In both enzymes, the outer surface was slightly negative, with a roughly neutral patch surrounding the highly positive inner surface of the β-barrel, which indeed bound the negatively charged FMN molecule. Given the high sequence identity, similar predicted 3D folding, and surface electrostatics, the very different substrate spectrum observed with these two enzymes was likely to depend on a few relevant changes at the substrate binding site.

## 3. Materials and Methods

### 3.1. Organisms and Culture Conditions

The strain *Aspergillus niger* CBS 513.88 was purchased from the CBS (Centraalbureau voor Schimmelculture; Central Bureau of Fungal Cultures) strain collection (The Netherlands). The cells were grown on a solid malt extract broth medium (Sigma Aldrich, Milano, Italy) at room temperature for 3 days.

### 3.2. Sequence Analysis and Cloning

The NCBI database was used for DNA sequence analysis. Searches and multiple alignments of fungal OYE sequences were performed using tBLASTn [33] and Clustal Omega [34], respectively. The genomic DNA of the fungus *Botryotinia fuckeliana* B05.10 was kindly provided by Professor Paul Tudzynski from Münster University, while *Aspergillus niger* genomic DNA was extracted in house following an established protocol [35]. The cloning strategy for *Bf*OYE4 was reported elsewhere [19]. Similarly, the other coding sequences were obtained by PCR amplifications from genomic DNA, introducing the restriction sites NdeI and BamHI (HindIII for *An*OYE8) at the 5′ and 3′ ends of the open reading frames, respectively. The sequence of *Bf*OYE1 was also mutagenized in order to suppress a predicted N-glycosylation site (in light of its eventual expression in yeast), while for *An*OYE8, an additional PCR reaction was performed in order to remove an intronic sequence. The sequences of the synthetic oligonucleotides used for PCR amplifications are reported in Table S6. The expression vectors were produced by digestion of pET-28a(+) (Novagen, San Diego, CA, USA) with NdeI/BamHI (HindIII for *An*OYE8) (New England Biolabs, Ipswich, MA, USA) and ligation of the amplified OYE sequences cut by the same enzymes.

### 3.3. Production, Analysis, and Purification of Recombinant Proteins

The recombinant enzymes were produced in different strains of *E. coli* BL21 (DE3) (Novagen, San Diego, CA, USA) (see Table S7 for optimized conditions). Pre-cultures were carried out in a lysogeny broth medium (LB) (50 mL) containing kanamycin (50 μg/mL) at 37 °C. Preparative cultures were carried out in LB medium (1 L), and the cells were grown in a shaking incubator (180 rpm) at 37 °C to an optical density at 600 nm (OD_600_) of 0.4–0.6. Then, riboflavin (25–100 μM final concentration) and isopropyl-β-D-1-thiogalactopyranoside (IPTG, 0.2 mM final concentration) were added. After induction, the cells were cultured at the optimum temperature overnight (Table S7). The cells were harvested by centrifugation (4 °C, 10 min, 5000× *g*) and washed with Tris-HCl buffer (pH 8.0, 50 mM). Cell disruption was obtained by a French Press (Constant Systems Cell Disruptor OneShot; Constant Systems, Kennesaw, GA, USA), and crude extract was centrifuged (4 °C, 30 min, 18,000× *g*) to separate the soluble and insoluble fractions. To enhance flavination, FMN cofactor (at a 100-μM final concentration) was added to the crude extract before cell disruption. Synthesis of the recombinant proteins was checked by SDS-PAGE. Overexpressed proteins were purified by immobilized metal affinity chromatography (IMAC). The soluble fractions obtained from 1 L culture were incubated with Ni-NTA resin (Sigma Aldrich, Milano, Italy) for 30 min at 4 °C and then loaded and packed in a 10-mL empty Poly-prep^®^ column (Bio-Rad, Milano, Italy). The column was washed by gravity flow with five column volumes of Tris-HCl buffer (pH 8.0, 50 mM). Elution was performed by five column volumes of Tris-HCl (pH 8.0, 50 mM) and imidazole solution (250 mM). Alternatively, proteins were purified by fast protein liquid chromatography (FPLC) using a GE ÄKTA Purifier 100 FPLC System w/ UPC-900 (HisTrap™ High Performance 1-mL pre-packed columns (Cytiva, Global Life Sciences Solutions, Marlborough, MA, USA)). The soluble fraction was eluted twice and washed with 10 column volumes with Tris-HCl buffer (pH 8.0, 50 mM) and imidazole (10 mM). Elution was performed by a 0–100% gradient in 40 min (flow 1 mL/min). For the crystallization trials, an additional step of purification by size exclusion chromatography (SEC) was performed.

The concentration of the enzyme preparations was evaluated by spectrophotometric measurement of the concentration of free flavin in a solution of thermal denatured protein and calculated as previously reported [36].

### 3.4. Oligomeric State Determination by Analytical Gel Filtration and BN-PAGE

Analytical size exclusion chromatography analysis of *Bf*OYE1, *An*OYE2, and *An*OYE8 (150 µg of purified protein) was performed with an ÄKTA purifier system using a Superdex 200 10/300 GL column (GE Healthcare, Milano, Italy). The matrix was equilibrated with Tris–HCl buffer (pH 8.0, 50 mM) and NaCl (150 mM). The flow rate was kept constant at 1 mL/min. The apparent Kr values of the eluted peaks were determined by comparison to a calibration curve obtained with four standard proteins—carbonic anhydrase (30 kDa and Kr 0.49), ovalbumine (45 kDa and Kr 0.43), bovine serum albumin (66.5 kDa and Kr 0.36), and ferritin (440 kDa and Kr 0.17)—as reported in Figure S3. BN-PAGE was performed by using NativePAGE™ 3 to 12%, Bis-Tris, 1.0 mm, Mini Protein Gels from Thermo Fischer Scientific (Waltham, MA, USA). Then, 12.5 µg of purified protein samples were run at 150 V for 2 h using NativePAGE™ Anode and Cathode Buffer Additives from Thermo Fischer Scientific (Waltham, MA, USA). Bovine serum albumin (BSA; monomeric species 66.5 kDa and dimeric species 133 kDa) and human serum albumin (HSA; monomeric species 66.37 kDa and dimeric species 132.74 kDa) (25 and 30 µg, respectively) were used for comparison of the MWs.

### 3.5. Activity Assay and Kinetics

ER activity was determined by monitoring the consumption of NADPH at 340 nm (ε = 6.22 mM^−1^ cm^−1^) using an Agilent 8453 or a Cary spectrophotometer against a range of activated alkenes. In the case of ketoisophorone (**4**), the assay was performed at 365 nm using a molar absorption coefficient of 3.51 mM^−1^ cm^−1^ [37]. The standard assay (100 μL) was performed at 25 °C in Tris–HCl buffer (pH 8.0, 50 mM) containing NADPH (100 μM) and the substrate (10 mM) dissolved in 100% ethanol (1% final concentration). The reaction was started by adding the enzyme to the 200-nM final concentration. One unit of ER activity is defined as the amount of protein that reduces 1 μmol of NADPH per minute. The steady state kinetic parameters of the different substrates were determined using substrate concentrations ranging from 0 to 25 mM. The data were fitted using the Michaelis–Menten equation in the program Graph-Pad Prism v5.0 (GraphPad Software, San Diego, CA, USA).

### 3.6. Determination of pH Optimum

For the determination of the pH optimum, the specific activities (U/mg) were evaluated in a universal buffer of a constant ionic strength (AcOH 50 mM, MES 50 mM, Tris 50 mM, and CAPS 50 mM) adjusted to the desired pH values (4–11) at 25 °C using either NaOH or HCl. The standard assay (100 μL) was performed using NADPH (100 μM), maleimide (**5**) (for *An*OYE2 and *An*OYE8) (10 mM), and cyclohex-2-en-1-one (**2**) (for *Bf*OYE1) as standard substrates. The reactions were started by the addition of purified enzymes (200 nM) and monitored over 1 min.

### 3.7. Thermofluor Measurements

The apparent unfolding temperatures of the recombinant enzymes T_m_ in standard conditions (Tris, pH 8.0, 50 mM) and in the presence of different co-solvents (ethanol, acetone, acetonitrile, dimethyl sulfoxide, and dioxane) at different percentages (5–50% *v*/*v*) were determined using the Thermofluor method as previously described [36]. All the purified proteins were used and diluted to 5 μM in Tris buffer (pH 8.0, 50 mM). All measurements were performed in triplicate.

### 3.8. Crystallization and Data Collection

Recombinant *An*OYE8 (15 mg/mL in Tris-HCl buffer, pH 8.0, 50 mM) was screened by high-throughput sparse matrix crystallization trials and dispensed by Oryx8 Robot (Douglas Instruments, EastGarston, UK). MRC two-drop 96-well standard plates were adopted both in the initial screenings and the following optimization steps. All the conditions were deposited and left equilibrating by vapor diffusion at 293 K. A panel of 288 crystallization conditions was tested (PACT, LMB, and MORPHEUS screens, Molecular Dimension Ltd., Sheffield, UK). Flat crystals of N-terminal His_6_-tagged *An*OYE8 appeared after one night of incubation. The most regular ones were grown in LMB screen n.69: PEG 5000 MME (18% *w*/*v*), MES (pH 6.5, 0.1 M), and ammonium sulphate (0.2 M). The X-ray diffraction data were collected at the ESRF (Grenoble, France) synchrotron radiation source (for beamline and data collection details, see Table S4).

### 3.9. Model Building and Refinement

All the diffraction data were processed and analyzed by the automated pipelines feasible at the ESRF synchrotron in Grenoble. In particular, for *An*OYE8, the data were integrated and scaled by the XDSAPP autoprocessing framework [38]. The obtained data were further cut to an appropriate resolution by running Aimless with the CCP4i2 suite [39]. The same interface was used in combination with Phenix suite for any of the subsequent steps of phasing and refinement. The *An*OYE8 structure was determined by molecular replacement using as a template a model of the *An*OYE8 enzyme, built by a Swiss model server [40] (PDB 5LNJ was used as a template). The refinement steps were carried out by Refmac5 [41] and Phenix software Refine [42]. Four molecules per asymmetric unit and roughly 45% of the solvent defined the crystal content. The final model was traced and visible from Asp3 to Lys416 in the E and G chains and from Asp3 to Pro417 in the B and C chains, with some gaps between residues 402–404 for chains C, B, and E, residues 404–405 for chain G, between residues 276–278 in chain G, and residues 277–281 in chain B. Flavin cofactor FMN was automatically imported from the Coot dictionary and fitted by a ligand search run. FMN cofactor was easily placed and clearly defined in each of the four molecules present in the asymmetric unit. The final parameters obtained for the best dataset (2.8 Å) reached an Rfactor/Rfree ratio of 0.24/0.26. Structure analysis was performed by PISA [31] and tools feasible in the ccp4i2 package [39].

### 3.10. Bioinformatic Analyses

The *Bf*OYE1 and *An*OYE2 models were modeled at the SwissModel server [40] using the EasA crystal structure (PDB 4NQW) as a template. The model quality was assessed via QMEAN6, QMEANDisCO, and MolProbity [43,44], and the protonation state at pH 8.0 was assigned with PDB2PQR using the CHARMM forcefield [45]. Energy minimization was performed via Gromacs 2021.3 [46] to optimize the side chain packing and interactions. Specifically, the CHARMM36 forcefield [47] was used, and the system energy was minimized by 5000 steps of the steepest descent energy minimization with a tolerance of 1000 kJ/mol/nm. FMN was docked to minimized structures using COFACTOR [48] at the Zhang lab server, and the active site properties were evaluated through the CASTp 3.0 server [49]. Electrostatic maps were obtained via APBS [50] and plotted at ±7 kBT/e using UCSF Chimera 1.15 [51]. Specifically, the dielectric constant values for the protein interior (εp) and solvent (εs) were set as εp = 2 and εs = 78.54 [52,53,54]. The probe radius for the dielectric surface and ion accessibility surface were set as r = 1.4 Å and r = 2.0 Å, respectively, and calculations were run at 310 K for simulating 150 mM NaCl.

## 4. Conclusions

Since the isolation of the first yellow enzyme from *Saccharomyces pastorianus* in 1932 by Warburg and Christian, the superfamily of Old Yellow Enzymes has gradually grown, reaching a number of members that is now close to a hundred. Even though the native functionalities of these enzymes are still mostly elusive, they were successfully applied in the asymmetric reduction of a broad variety of “synthetic” substrates, and more recently, reactivities other than the stereospecific addition of hydride to C=C bonds were described.

In general, modeling and structural studies coupled to biochemical characterization represent key elements for guiding protein engineering and, ultimately, the selection of the most appropriate biocatalyst in a peculiar synthetic process. Our study adds four new members to this wide family, providing biochemical and structural details. The catalytic proficiency was determined for a panel of representative substrates in the context of activated C=C double bond reduction catalysis. The enzymes in our hands showed a robust activity toward maleimide and an exquisite dependence from NADPH as an electron donor, except for *An*OYE8. The latter refractory behavior toward this and other standard substrates can be partially justified by its structural features. Indeed, the crystal structure of *An*OYE8 was described here for the first time, and it was very similar to the one of *Bf*OYE4 as previously determined by our group [19]. This revealed a peculiar small catalytic pocket deeply shaped by the dimeric organization and the very long C-term and N-term extensions of both protomers. On the other hand, in agreement with the spectrum of substrates tested and the reduction activity observed in this preliminary characterization, the *An*OYE2 and *Bf*OYE1 structure models suggest a larger and more promiscuous catalytic region that seems to distinguish these enzymes from other “classical” OYEs, populating the Class II branch of the OYE tree.

As for the source of these enzymes, filamentous fungi share with bacteria the proscenium of the phylogenetic classification, which is constantly updated. Considering their ecological biodiversity, metabolic versatility, and provision of a number of different and specific isoforms, filamentous fungi represent a real spring of information for expanding the catalytic potentialities and enlarging the toolbox of OYEs. The chemical and pharmaceutical industries are constantly looking at this wide family to select “in direct line” members or “in-law” (acquired by engineering) relatives to make processes involving stereoselective C=C bond reductions more convenient and greener than the present ones.

## Figures and Tables

**Table 1:** Spectrophotometric substrate screening ^1^.

	Specific Activity (U/mg)			
Substrate	*An*OYE2	*An*OYE8	*Bf*OYE1	*Bf*OYE4
O_2_	0.37 ± 0.07	0.28 ± 0.07	0.32 ± 0.00	0.17 ± 0.00
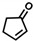 **1**	N.D.	1.1 ± 0.08	3.8 ± 0.22	2.2 ± 0.19
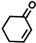 **2**	N.D.	0.7 ± 0.08	10.3 ± 0.00	3.3 ± 0.00
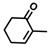 **3**	N.D.	0.8 ± 0.08	5.7 ± 0.37	1.4 ± 0.31
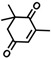 **4**	N.D.	N.D.	10.3 ± 0.30	N.D.
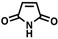 **5**	16.6 ± 0.53	N.D.	11.3 ± 0.30	22.5 ± 0.25
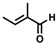 **6**	1.5 ± 0.07	1.0 ± 0.00	7.5 ± 0.60	0.9 ± 0.06
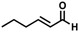 **7**	N.D.	1.0 ± 0.08	9.4 ± 0.50	0.8 ± 0.13
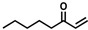 **8**	N.D.	1.2 ± 0.00	4.7 ± 0.15	23.4 ± 1.87

^1^ The standard assay (100 μL) was performed at 25 °C in Tris-HCl (pH 8.0, 50 mM) containing NADPH (100 μM) and the substrate (10 mM). The reaction was started through the addition of the enzyme to a final concentration of 200 nM. All measurements were performed in triplicate. Background oxidase activity was measured in the absence of substrates (first row; O_2_), but it was not subtracted from the specific activity for the other substrates. N.D. = not detected.

**Table 2:** Steady state kinetic parameters of *An*OYE2, *Bf*OYE1, and *Bf*OYE4 ^1^.

Substrate	*An*OYE2			*Bf*OYE1			*Bf*OYE4		
	K_M_ (mM)	k_cat_ (s^−1^)	k_cat_/K_M_ (mM^−1^ s^−1^)	K_M_ (mM)	k_cat_ (s^−1^)	k_cat_/K_M_ (mM^−1^ s^−1^)	K_M_ (mM)	k_cat_ (s^−1^)	k_cat_/K_M_ (mM^−1^ s^−1^)
**NADPH ^2^**	0.037 ± 0.01	8.00 ± 0.40	216.2	0.025 ± 0.00	5.83 ± 0.35	233.2	0.13 ± 0.02	89.22 ± 3.37	686.3
**1**	N.M.	N.M.	N.M.	2.82 ± 0.36	5.98 ± 0.21	2.1	1.07 ± 0.12	1.06 ± 0.03	1.0
**2**	N.M.	N.M.	N.M.	0.44 ± 0.06	5.00 ± 0.16	11.4	0.65 ± 0.09	2.57 ± 0.07	3.9
**3**	N.M.	N.M.	N.M.	0.44 ± 0.06	4.13 ± 0.12	9.4	1.83 ± 0.18	0.56 ± 0.013	0.3
**4**	N.M.	N.M.	N.M.	2.14 ± 0.28	6.32 ± 0.28	2.9	N.M.	N.M.	N.M.
**5**	0.01 ± 0.00	7.38 ± 0.66	738.0	0.01 ± 0.00	9.91 ± 0.29	991.0	0.07 ± 0.01	36.3 ± 1.12	518.6
**6**	N.M.	N.M.	N.M.	0.07 ± 0.01	7.84 ± 0.01	112.0	N.M.	N.M.	N.M.
**7**	N.M.	N.M.	N.M.	0.45 ± 0.06	10.6 ± 0.47	23.6	N.M.	N.M.	N.M.
**8**	N.M.	N.M.	N.M.	N.D.	N.D.	N.D.	2.72 ± 0.50	22.6 ± 1.14	8.3

^1^ The standard assay (100 μL) was performed at 25 °C in Tris-HCl (pH 8.0, 50 mM), NADPH (100 μM), and ERs (200 nM, except for substrate **5**, where 40 nM *Bf*OYE1 and 20 nM of *An*OYE2 and *Bf*OYE4 were used). N.D. = not detected; N.M. = not measured. ^2^ The standard assay (200 μL) was performed at 25 °C in Tris-HCl (pH 8.0, 50 mM), maleimide (**5**) (1 mM), and ERs (20 nM).

## Data Availability

Not applicable.

## Supplementary Materials

The following supporting information can be downloaded at https://www.mdpi.com/article/10.3390/ijms23063050/s1. Reference [55] is cited in the supplementary materials.
