# Supplementary material for: The Family Keeps on Growing: Four Novel Fungal OYEs Characterized
Source: Int J Mol Sci. 2022 Mar 11;23(6):3050. doi: 10.3390/ijms23063050 (PMC8954901; doi:10.3390/ijms23063050)
Supplement: Supplementary file 1 [file ijms-23-03050-s001.zip › ijms-1600422-supplementary.pdf]

SUPPORTING INFORMATION

to the article

The family keeps on growing: four novel fungal OYEs characterized

Marina Simona Robescu [1,#], Giovanni Loprete [1], Matteo Gasparotto [1], Filippo Vascon, [1] Francesco Filippini [1], Laura Cendron [1] and Elisabetta Bergantino [1,*]

[1] Synthetic Biology and Biotechnology Unit, Department of Biology, University of Padova, viale G. Colombo 3, I-35131 Padova, Italy

[#] Present address: Department of Drug Sciences, University of Pavia, viale Taramelli 12, 27100 Pavia, Italy

*Corresponding author: elisabetta.bergantino@unipd.it

Table of contents

Supplementary Figures

Figure S1. Sequence alignment of the four putative ERs with representative OYEs from different classes

Figure S2. SDS-PAGE analysis of *An*OYE2, *An*OYE8 and *Bf*OYE1 purification in the optimized conditions

Figure S3. SDS-PAGE analysis of *Bf*OYE1 synthesis with and without the addition of riboflavin precursor

Figure S4. SDS-PAGE analysis of *An*OYE2 synthesis in different *E. coli* strains

Figure S5. SDS-PAGE analysis of *An*OYE8 synthesis in different *E. coli* strains

Figure S6. Determination of purified *An*OYE2, *An*OYE8 and *Bf*OYE1 concentration

Figure S7. Calibration curve of Superdex 200 10/300 GL column

Figure S8. Protein activity-pH profiles

Figure S9. Influence of different types and different percentages of organic co-solvents on T_m

Figure S10. Schematic illustration of the interaction of FMN with active site residues of *An*OYE8

Figure S11. Superposition of *An*OYE8 and *Bf*OYE4 crystal structures

Figure S12. Comparison of *An*OYE8 dimer and *Bf*OYE4 dimer active site cavity architecture

Figure S13. Comparative analysis of surface charge distribution between members of Class III OYEs

Figure S14. Model structures of *An*OYE2 and *Bf*OYE1

Figure S15. Schematic illustration of the interaction of FMN with active site residues of *An*OYE2

Figure S16. Schematic illustration of the interaction of FMN with active site residues of *Bf*OYE1

Figure S17. Comparison of loops among Class II homologues crystal structures

Figure S18. Pseudo-atom distances between the 'bounding' residues in Class II homologues

Supplementary Tables

Table S1. List of putative OYEs translated from the genomes of *Aspergillus niger* and *Botryotinia fuckeliana*

Table S2. List of OYE sequences used for phylogenetic analysis and their accession numbers

Table S3. Comparison of *An*OYE8 and *Bf*OYE4 characteristics with those of other Class III OYEs

Table S4. X-ray crystallographic data collection and refinement statistics for *An*OYE8

Table S5. Quality of *An*OYE2 and *Bf*OYE1 models

Table S6. Oligonucleotides used for cloning and sequencing of OYE ORFs

Table S7. Optimized expression conditions for *An*OYE2, *An*OYE8 and *Bf*OYE1 recombinant production

References

```
OYE1        ------------------------------MSFV---------KDFKPQ--ALGDTNLFK   19
YqjM        -----------------------------------------------------MARKLFT   7
Ppo_ER3     ----------------------------------------------------MNTELLFK   8
YqiG        --------------------------------------------------MNPKYKPLFE   10
AnOYE2      -----------------------------------------------------MCSKLFQ   7
AnOYE8      -------------------MKDIKVEPAKGISYFTPAQETPAGTAANPQTSGKAIPKLFQ   41
BfOYE1      ----------------------------------------------------MSPSTLFT   8
BfOYE4      MSTPTPHPQHGVPCGTSTPRPGLLNTPAPGVPFYTPLQSPPSGTALHL---SPSTPKLFT   57
                                                                     **

OYE1        PIKIG-NNELLHRAVIPPLTRMRALHPGNIPNRDWAVEYYTQRAQRPGTMIITEGAFISP   78
YqjM        PITIK-DMTLKNRIVMSPMCMYSSHEK-DGKLTPFHMAHYISRAIGQVGLIIVEASAVNP   65
Ppo_ER3     PFKAG-NLSLPNRIVMAPMTRNFSPQ---GIPGPEVAAYYRRRAENAVGLIITEGTAINH   64
YqiG        PFTFKSGVTINNRIAVAPMTHYASN-E-DGTISEAELD-YIIPRSKEMGMVITACANVTP   67
AnOYE2      PLQIG-KTNLEHRVVMAPLTRFRADSQ--HVPLPMATTYYEQRASVPGTLLIAEATLISP   64
AnOYE8      PITIR-GLTFQNRLGVSPMCQYSA--E-DGHMTDYHLAHLGGIAQRGPGLIMIEATAVQP   97
BfOYE1      PLKVG-TSELQHRIAMAPLTRFRADDN--HVPLPMVAEYYAQRASVPGTLLVSEATFIAP   65
BfOYE4      PLKIR-SLTLQNRIMLSPMCQYSA--S-NGHFTPWHMAHLGGIISRGPGLSMVEATSVLP   113
            *:       : :*  : *:    :                         : :   : :

OYE1        QAGGYDNAPGVWSEEQMVEWTKIFNAIHEKKSFVWVQLWVLGWAAFPDNLA---------   129
YqjM        QGRITDQDLGIWSDEHIEGFAKLTEQVKEQGSKIGIQLAHAGRKAELEG-----------   114
Ppo_ER3     PAAVEHTSIPNFYGEGLEGWAKVVEEVHAVGGKIIPQLWHVGTARKIGADN---------   115
YqiG        DGKAFPGQPAIHDDSNIPGLKKLAQAIQAQGAKAVVQIHHGGIECPSELVP---------   118
AnOYE2      SAGGVPHAPGVWSEEQVNGWKKITEAVHKKGSFIFCQLIAAGRAADPAQLH---------   115
AnOYE8      EGRISPQDVGLWKDSQIAPIARVIEFAHSQGQKIGIQLAHAGRKASTTVPWMLNHGSIAT   157
BfOYE1      RAAGYANPPGIWNKEQIAGWKKVTDAVHAKKSYIWMQLWALGRAADPSVLQ---------   116
BfOYE4      EGRITPEDSGLWLDSQGDKLKEVVQFAHSQGQLIGIQLSHAGRKASMVAPWLD-RSAVAT   172
             .            .      .: :  :        *:   *

OYE1        -RDGLR--YDSASDNVFMDAEQEAKAKKANNPQHSLTKDEIKQYIKEYVQAAKNSIAAGA   186
YqjM        --------DIFAPSAIAFD--------EQSATPVEMSAEKVKETVQEFKQAAARAKEAGF   158
Ppo_ER3     -Q----------PNPEALPVGPSGISPAGEKVVEPLTEAEIADIISAYAQAAADAQRVGF   164
YqiG        ------QQDVVGPSDV-FD--------NGKQIARALTEEEVENIVKAFGEATRRAIEAGF   163
AnOYE2      -AEGGF--MLHAPSPIPIE--------PGMPVPKELDEIEVQEIINDFAMAAKNAIRAGF   164
AnOYE8      ENVGGWPDNVKGPSDIPFS--------ETFPRPRAMTQDDIREFKEAWVAAAKRALVAGA   209
BfOYE1      -QEGGY--KLQSSSDIAFE--------GG-GKPEPLTEAEIKEYIELYTQAAKNAIEAGF   164
BfOYE4      EEAGGWPTKVKGPSAIPYD--------EHHYKPSAMTLEDIQEFKDAWAASLKRALKAGF   224
                         .                     :   .: :  . :  :   :  .*

OYE1        DGVEIHSANGYLLNQFLDPHSNTRTDEYGGSIENRARFTLEVVDALVEAIGHEK-VGL--   243
YqjM        DVIEIHAAHGYLIHEFLSPLSNHRTDEYGGSPENRYRFLREIIDEVKQVW--DGPLFV--   214
Ppo_ER3     DGIELHGAHGYLIDQFFWDKTNKRTDQYGGNLVQRTRFAVEVIEACRRAVGPNFPIVL--   222
YqiG        DGVEIHGANGYLIQQFYSPKTNQRTDRWGGSDEKRLAFPLAIVDEVKKAASEHAKGAFLV   223
AnOYE2      DGVEIHGANGYLVDQFLQDVSNKRNDKWGGSIPNRARFGLEVAKAVADAIGADR-LGF--   221
AnOYE8      DFIEIHNAHGYLLASFLTPYANKRTDEYGGSFENRMRLPLKIAQLTRDTVGEHVPVFL--   267
BfOYE1      DGVEIHGANGYLIDQFFQDTANQRTDSWGGSVENRARFGLEVAKSVVAAVGAEK-TSM--   221
BfOYE4      DVIEIHNAHGYLLHEFVSPVSNKRTDQYGGSFENRIRLTLEIVEITRKIIPESMPLFL--   282
            * :*:* *:***: .*    :* *.* :**.  :*  :   : .             .

OYE1        --RLSPYGVFNSMSGGAET-GIVAQYAYVAGELEKRAKAGKRLAFVHLV-----EPRVTN   295
YqjM        --RVSASDYTDK------GLDIAD-HIGFAKWMKE-----QG-----VDLIDCSSGALVH   255
Ppo_ER3     --RFSQWKMYHYEEKLAQTPQELEQF---LTPLVK-----AGVDIFHCS-----SRRFWE   267
YqiG        GYRLSPEEP------ETPGLTMTE-TYTLVDALGD-----KELDYLHISLMDVNSKARRG   271
AnOYE2      --RLSPWNTWQSMKMV----DPVPQFTYFVERLQQ-----LGLAYLHVI-----ESRVIN   265
AnOYE8      --RLSASDWLGST--STETWDLQH-AVRFAEALAD-----QG----AIDLVDVSSGGLHS   313
BfOYE1      --RLSPFSPFQGMKMA----DPIPQFTYIAQELKK-----LNLAYLHVV-----ESRIIG   265
BfOYE4      --RISATDWLDYEGFGEESWTVAD-SARLAGILAD-----RG-----VDLMDVSSGANHP   329
              *.*                           : .                   .

OYE1        PFLTEGEGEYEGGSNDFVYSIWKG-----PVIRAGNFALH--------------------   330
YqjM        -ADINVFPGYQ---VSFAEKIREQ--ADMATGAVGMITDGSMAEEILQN-----------   298
Ppo_ER3     P-------EF--EGSDLNLAAWTKKITGKPVITVGSIGLEKAFLSDLEKNNNRQTDQSSS   318
YqiG        ADP-------T---RTRMDLLNERVGNKVPLIAVGSIHSADDALAVIE------------   309
AnOYE2      NVDCEK-----EGSIKFLLDVWGK---SAPVIVAGGYRPENV------------------   299
AnOYE8      SQEVKSGPGFQ---APFGIAVKKAVGERMLVATVGHIRDGKLANRLLEE-----------   359
BfOYE1      NADIEA-----TEKVDFLINIWNG---VSPILLAGGFTAESA------------------   299
BfOYE4      RQKITAGLGYQ---APFAKEIKRVVGERMLVGTVGMIGSGRQAEGLLSGMGGERG-----   381
                                             .*

OYE1        -----PEVVREEVKDKRTLIGYGRFFISNPDLVDRLEKG--LPLNK---YDRDTFYQM--   378
YqjM        --------------GRADLIFIGRELLRDPFFARTAAKQLNTEIPA--P---------VQ   333
Ppo_ER3     VEARLEQLVGQVEREEADLVAVGRALLVDPAFAVKLRDQQIEEIIP---YSDEVLKTL--   373
YqiG        --------------NGIPLVAMGREILVDPNWTVKVKEGREKQIETVIKGTDKEKYHLPE   355
AnOYE2      -----ENALEEEYKDYKVAVAFGRHFIANPDLPFRIRHD--IHLNP---YDRESFYTP--   347
AnOYE8      --------------EGLDVVLVGRGFQKDPGLVWTFAQHLDVEVAM--P---------GQ   394
BfOYE1      -----KKAVEEEYKGKDIVIVFGRYFITNPDLPFRVKEG--IEFTP---YDRDFFYNK--   347
BfOYE4      ---VDEGEEEEGKGTELDLVIVARGFQKNPGLVWEWAEELGVRIMV--A---------HQ   427
                               :  .* :  :*       .     .

OYE1        -------SAHGYIDY---PTYEEALKLGWDKK---      400
YqjM        YERGW------------------------------      338
Ppo_ER3     ------N----------------------------      374
YqiG        PLWQAIVNTQGWVPYKD------------------      372
AnOYE2      ------LQENGYTDY---PFSAEFIKSKTKC----      369
AnOYE8      IRWGFSKQGRRGTPFVDPSVYKPSVIE--------      421
BfOYE1      ------KEAEGYTTY---PFSKEFEAQRKAIESSA      373
BfOYE4      MRWGFRGKAGGH-----------------------      439
```

Figure S1. Sequence alignment performed by Clustal Omega of the four putative ERs from *Aspergilus niger* and *Botryotinia fuckeliana* with representative OYE homologues from different classes: OYE1 (Class II), YqjM (Class III), Ppo-ER3 (Class IV), YqiG (Class VI).

The catalytic residues are highlighted in red, the residues involved in FMN interaction are in orange, additional N-terminal residues in *An*OYE8 and *Bf*OYE4 are in blue. The finger print motifs reported by Oberdorfer et al.[1] are highlighted in pink for classical ERs and cyan for thermophilic-like ERs. The residues shaded in grey are highly conserved and the bold residues are similar. For thermophilic-like OYEs the residue involved in dimer formation is shown in cyan.

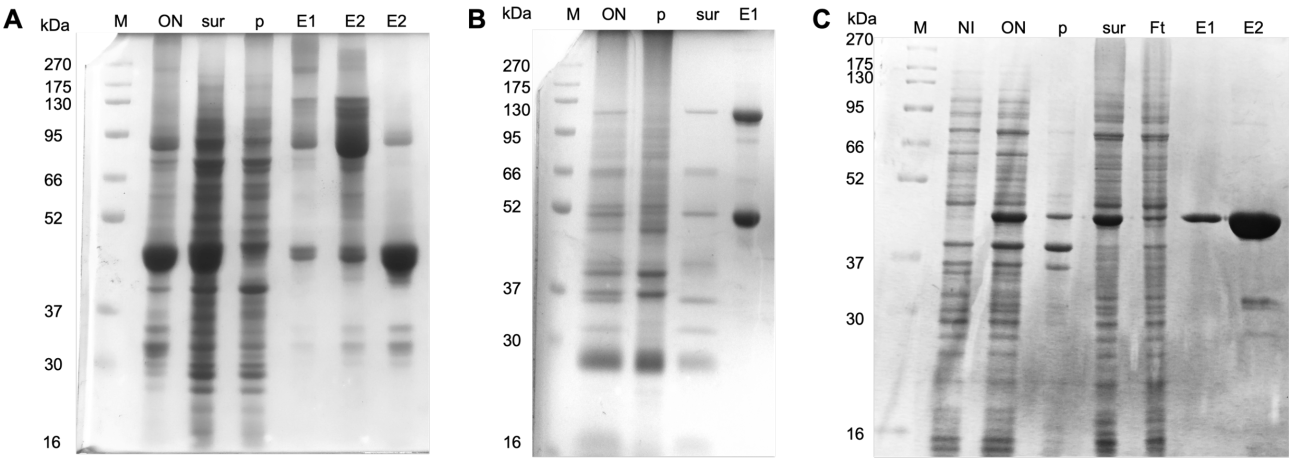

Figure S2. SDS-PAGE 12% acrylamide of *An*OYE2 (A), *An*OYE8 (B), *Bf*OYE1 (C) purification in the optimized conditions: Prestained SharpMassTM VII protein ladder (M), total cell extracts from non-induced (NI) and over-night induced cells with IPTG (ON), pellet fraction (p), soluble protein fraction (sur), flow-through (Ft), eluted fractions from IMAC (E1) and preparative SEC (E2).

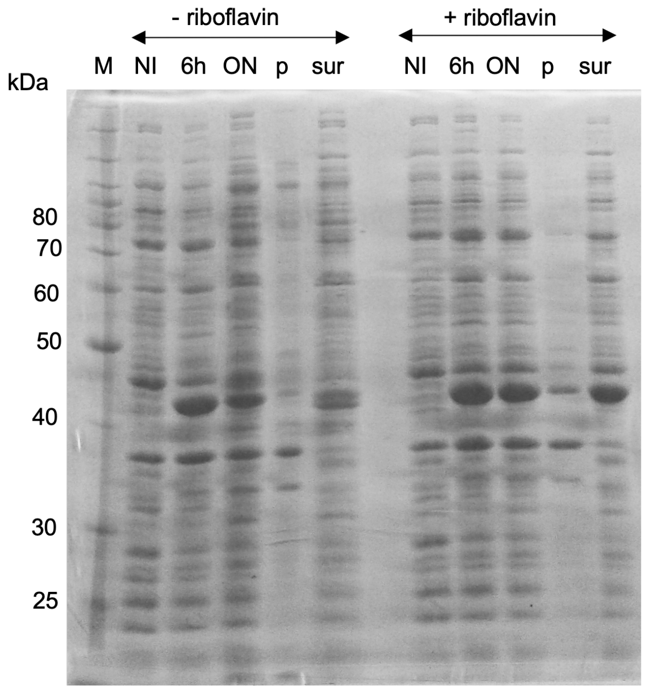

Figure S3. SDS-PAGE 12% acrylamide: BL21(DE3) grown at 25 °C expressing *Bf*OYE1 without and with the addition of riboflavin (25 µM): BenchMark™ protein ladder (M), total cell extracts from non-induced (NI) and 6 h and over-night induced cells with IPTG (6 h and ON), pellet fraction (p), soluble protein fraction (sur).

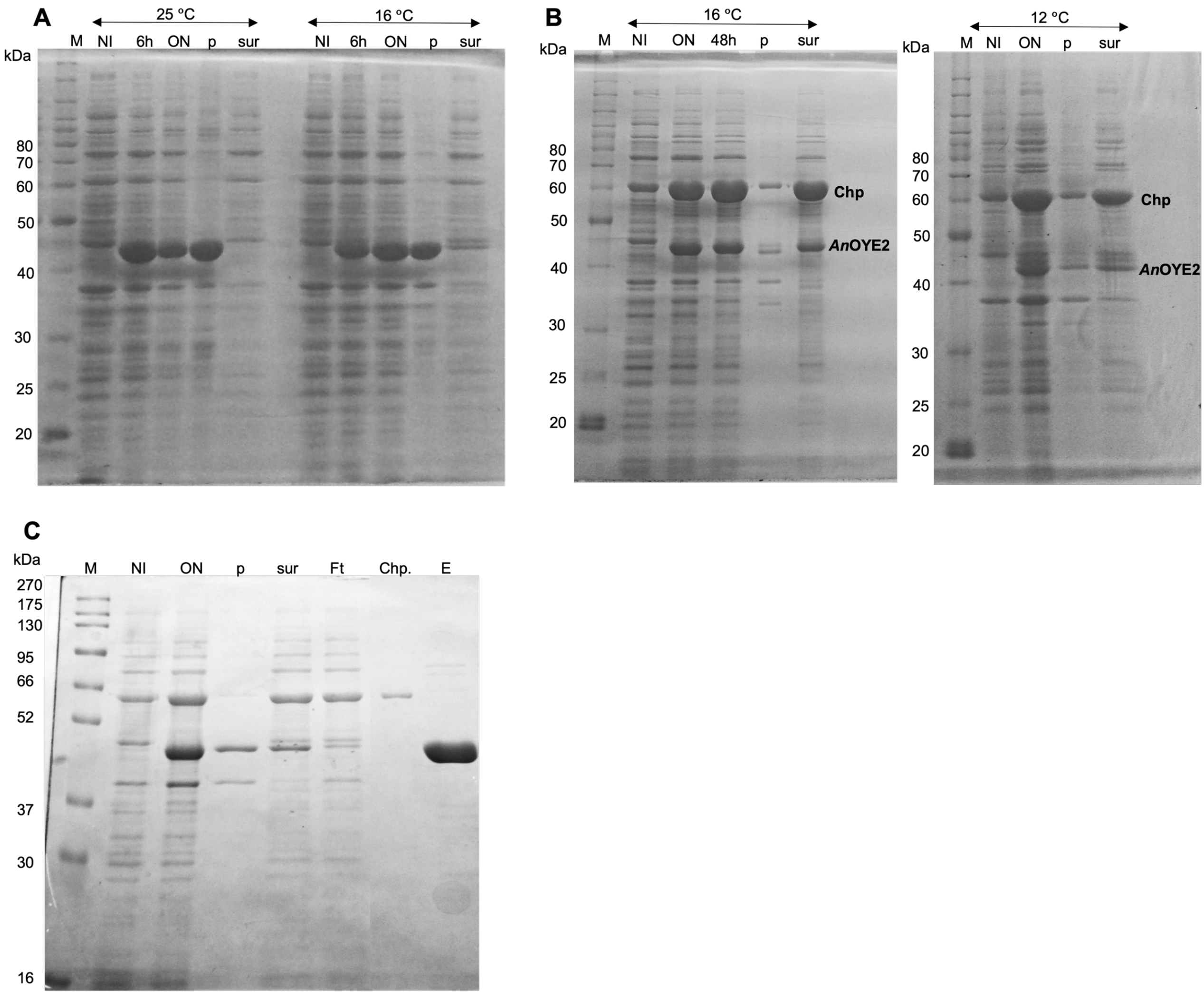

Figure S4. SDS-PAGE 12% acrylamide: *E. coli* BL21(DE3) grown at 25 °C and 16 °C (A) and *E. coli* BL21(DE3) Arctic® grown at 16 °C and 12 °C (B) expressing *An*OYE2: BenchMark™ protein ladder (M), total cell extracts from non-induced (NI) and 6 h, over-night and 48 h induced cells with IPTG (6 h, ON, 48 h), pellet fraction (p), soluble protein fraction (sur).
(C) SDS-PAGE 12% acrylamide of *An*OYE2 purification from *E. coli* BL21(DE3) Arctic® grown at 16 °C: Prestained SharpMass™ VII protein ladder (M), total cell extracts from non-induced (NI) and over-night induced cells with IPTG (ON), pellet fraction (p), soluble protein fraction (sur), flow-through (Ft), chaperonin removal (Chp.), eluted fractions from IMAC (E).

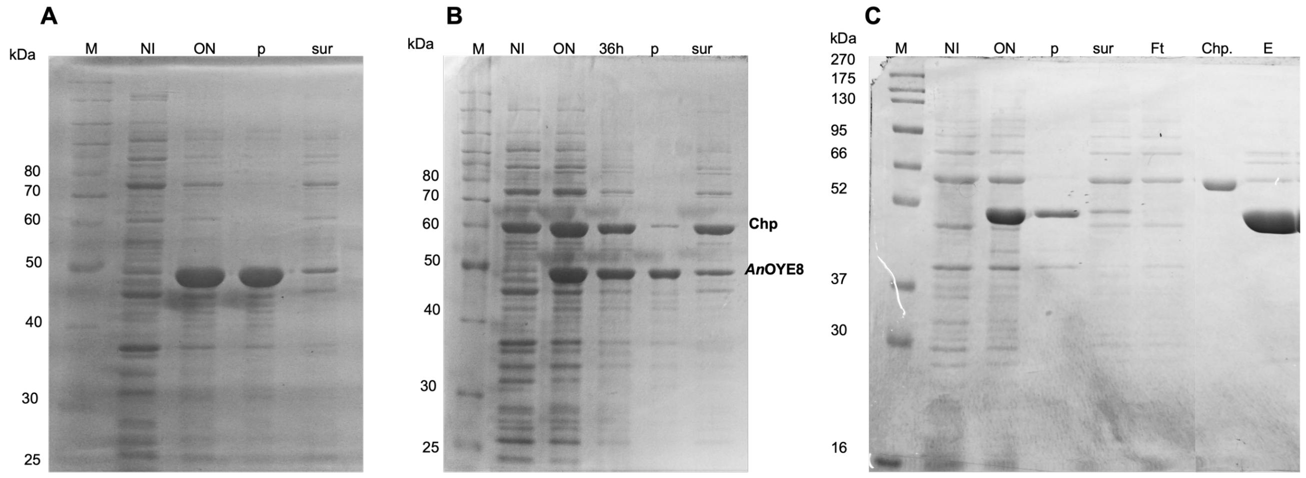

Figure S5. SDS-PAGE 12% acrylamide: *E. coli* BL21(DE3) grown at 25 °C (A) and *E. coli* BL21(DE3) Arctic® grown at 12 °C (B) expressing *An*OYE8: BenchMark™ protein ladder (M), total cell extracts from non-induced (NI) and over-night and 36 h induced cells with IPTG (ON and 36 h), pellet fraction (p), soluble protein fraction (sur).
(C) SDS-PAGE 12% acrylamide of *An*OYE8 purification from *E. coli* BL21(DE3) Arctic® grown at 12 °C: Prestained SharpMass™ VII protein ladder (M), total cell extracts from non-induced (NI) and over-night induced cells with IPTG (ON), pellet fraction (p), soluble protein fraction (sur), flow-through (Ft), chaperonin removal (Chp.), eluted fractions from IMAC (E).

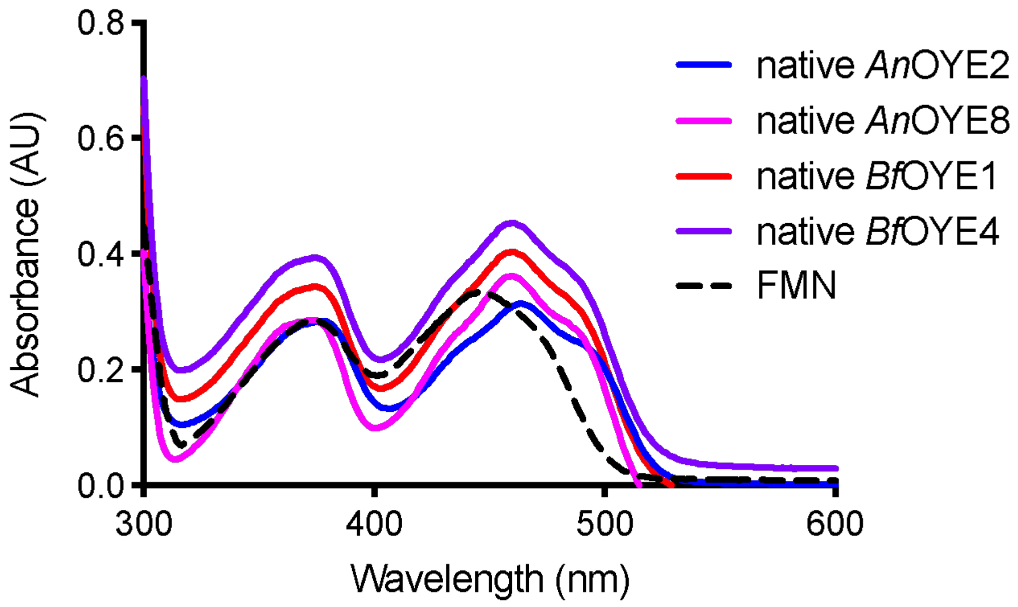

Figure S6. Determination of *An*OYE2, *An*OYE8 and *Bf*OYE1 concentration based on flavin absorption spectra of purified enzymes and released flavin after thermal denaturation (black dotted line).

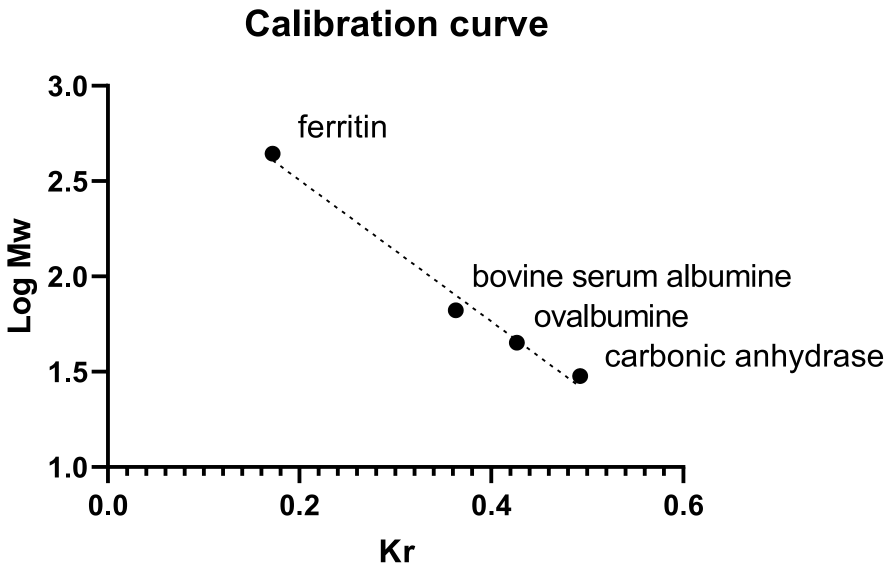

Figure S7. Calibration curve of Superdex 200 10/300 GL column (GE Healthcare) using standard proteins: carbonic anhydrase (30 kDa), ovalbumin (45 kDa), bovin serum albumin (66.5 kDa), ferritin (440 kDa).

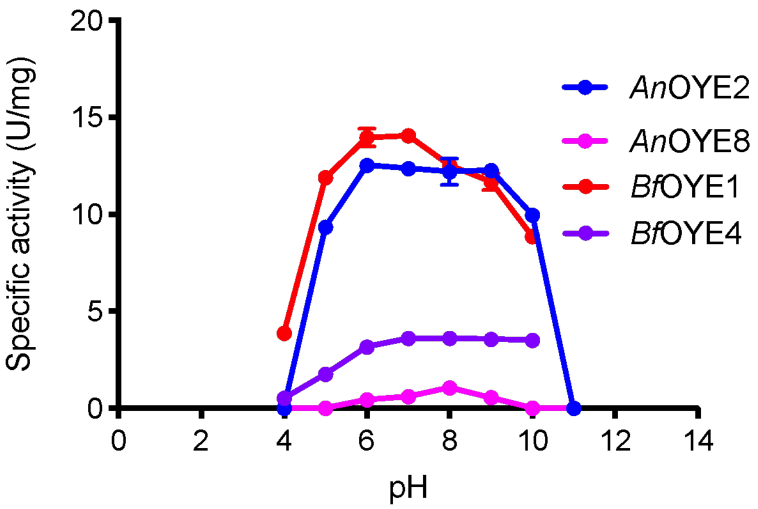

Figure S8. Protein activity-pH profiles: the specific activity (U/mg) was determined by monitoring the reduction of maleimide (**5**) (10 mM) (for *An*OYE2 and *An*OYE8) or cyclohex-2-en-1-one (**2**) (10 mM) (for *Bf*OYE1) in the presence of NADPH (100 mM). A universal buffer of constant ionic strength: AcOH (50 mM), MES (50 mM), Tris (50 mM) and CAPS (50 mM) adjusted to different pH values (4.0 - 11.0) at 25 °C was used. *Bf*OYE4 reported from Robescu et al.[2]

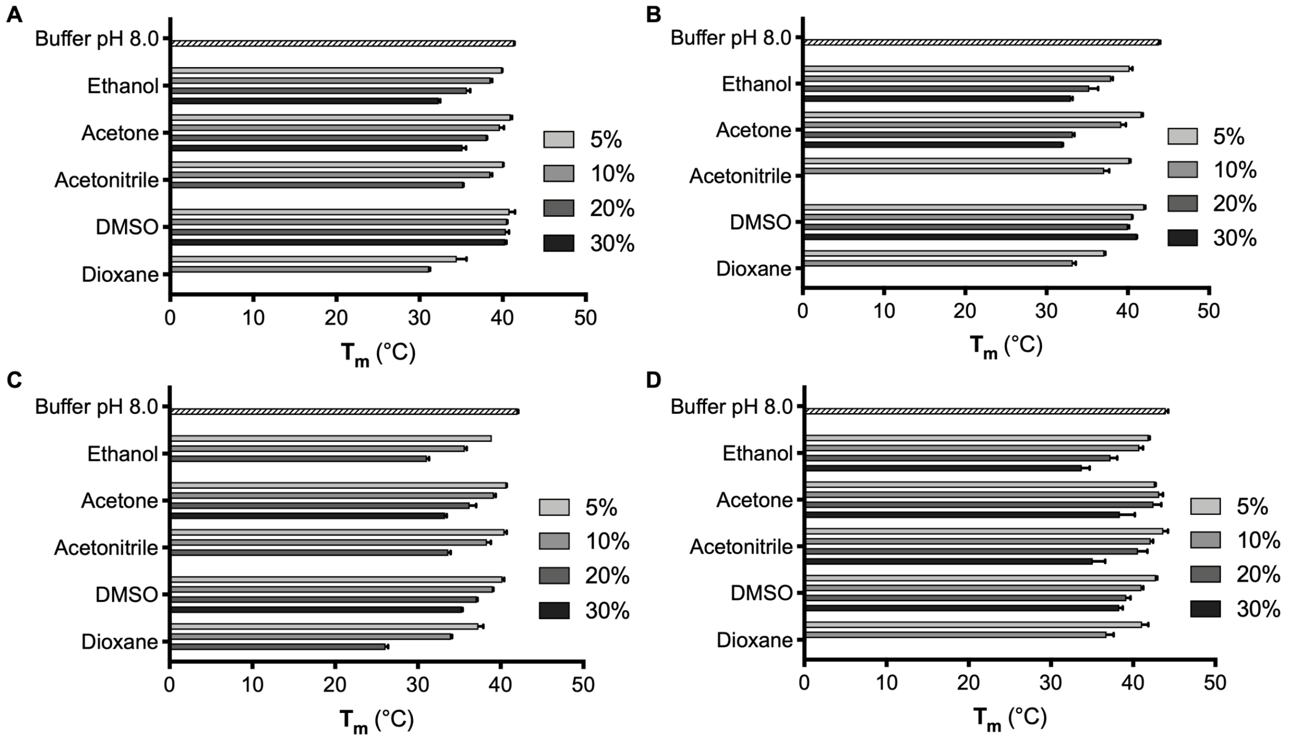

Figure S9. Influence of different types and different percentages of organic co-solvents (Tris-HCl buffer pH 8.0, 50 mM supplemented with increasing vol% of organic co-solvent) on proteins melting point T_m (°C) as measured by Thermofluor method. A) *An*OYE2; B) *An*OYE8, C) *Bf*OYE1; D) *Bf*OYE4.

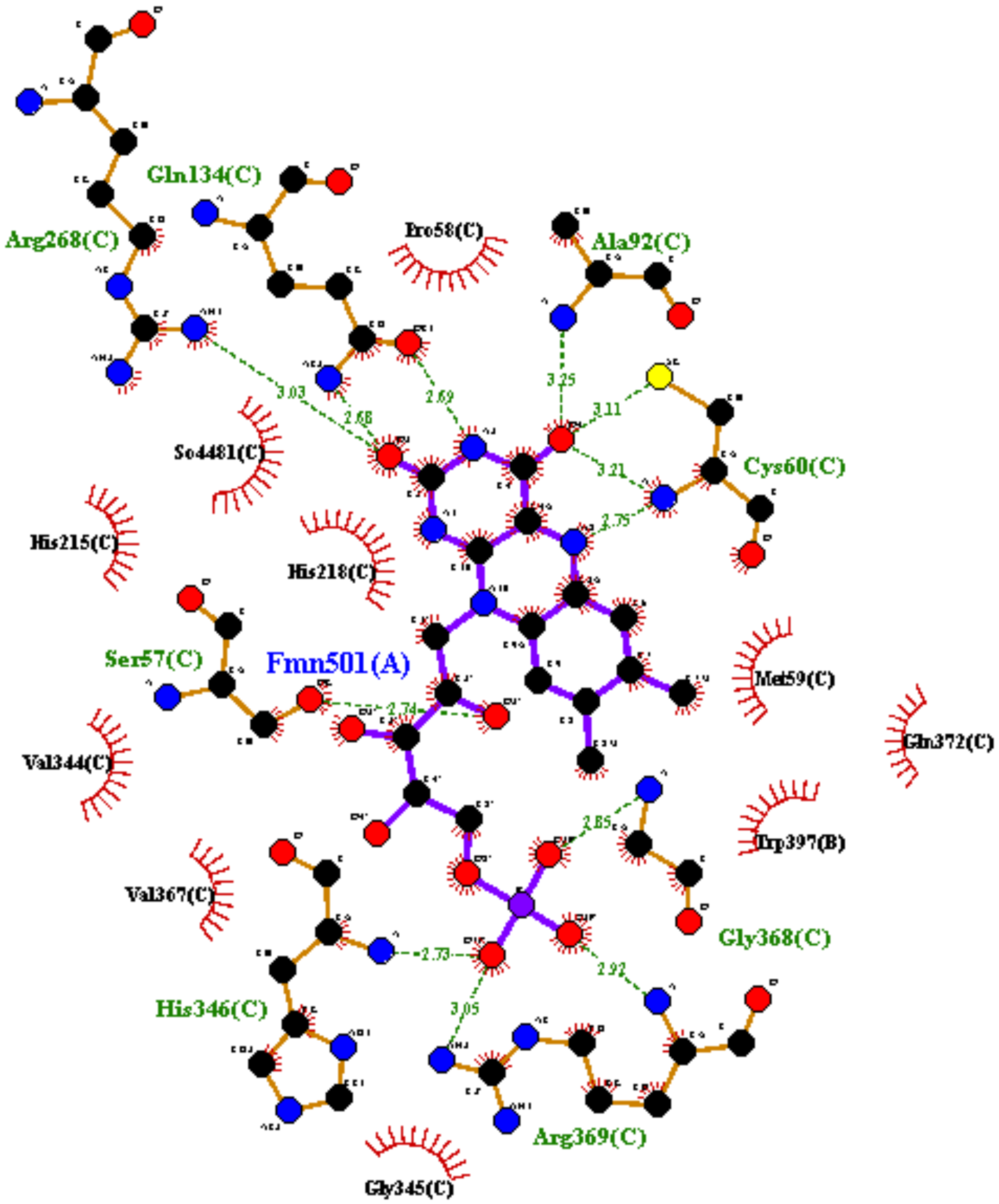

Figure S10. Schematic illustration of the interaction of FMN with active site residues of *An*OYE8. The thin green dotted lines illustrate hydrogen bonds.

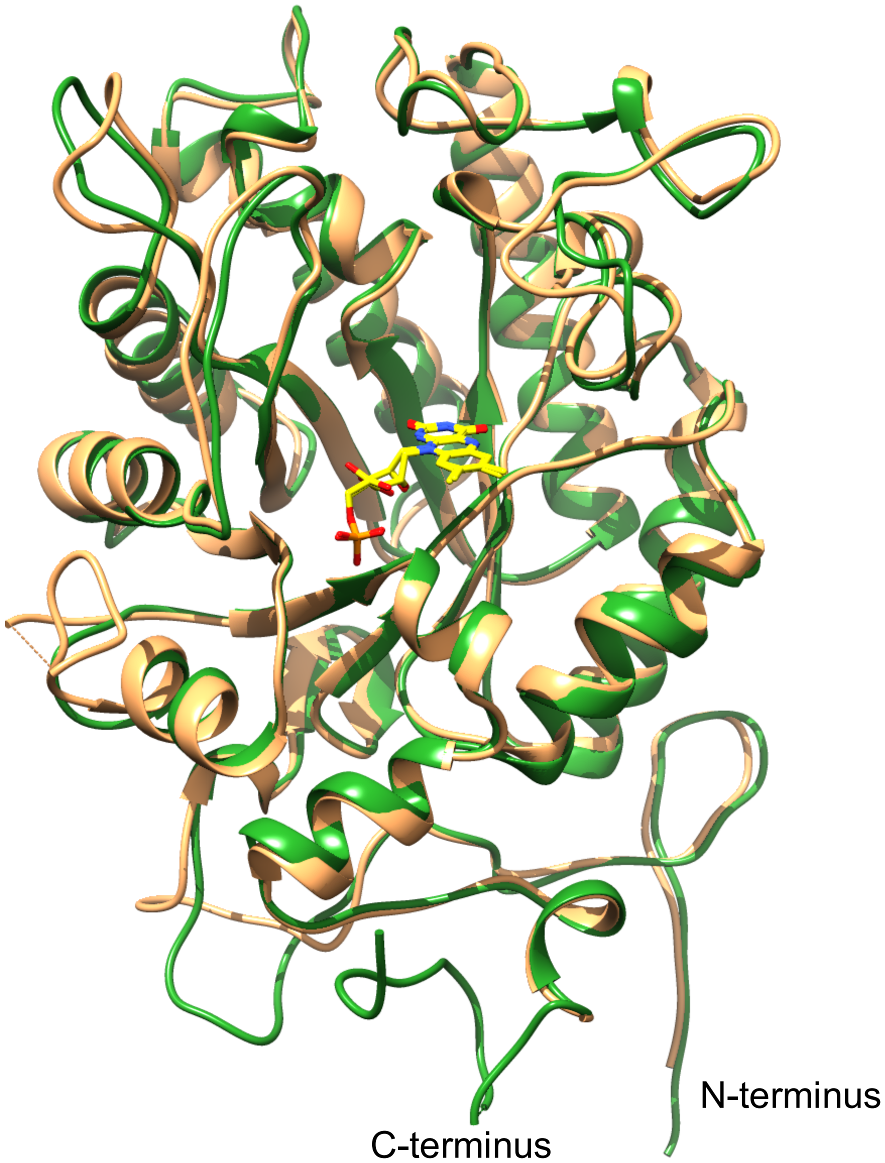

Figure S11. Superposition of *An*OYE8 (forest green) and *Bf*OYE4 (sandy brown) (pdb 7BLF) crystal structures. FMN cofactor bound in the active site is shown with C atoms in yellow.

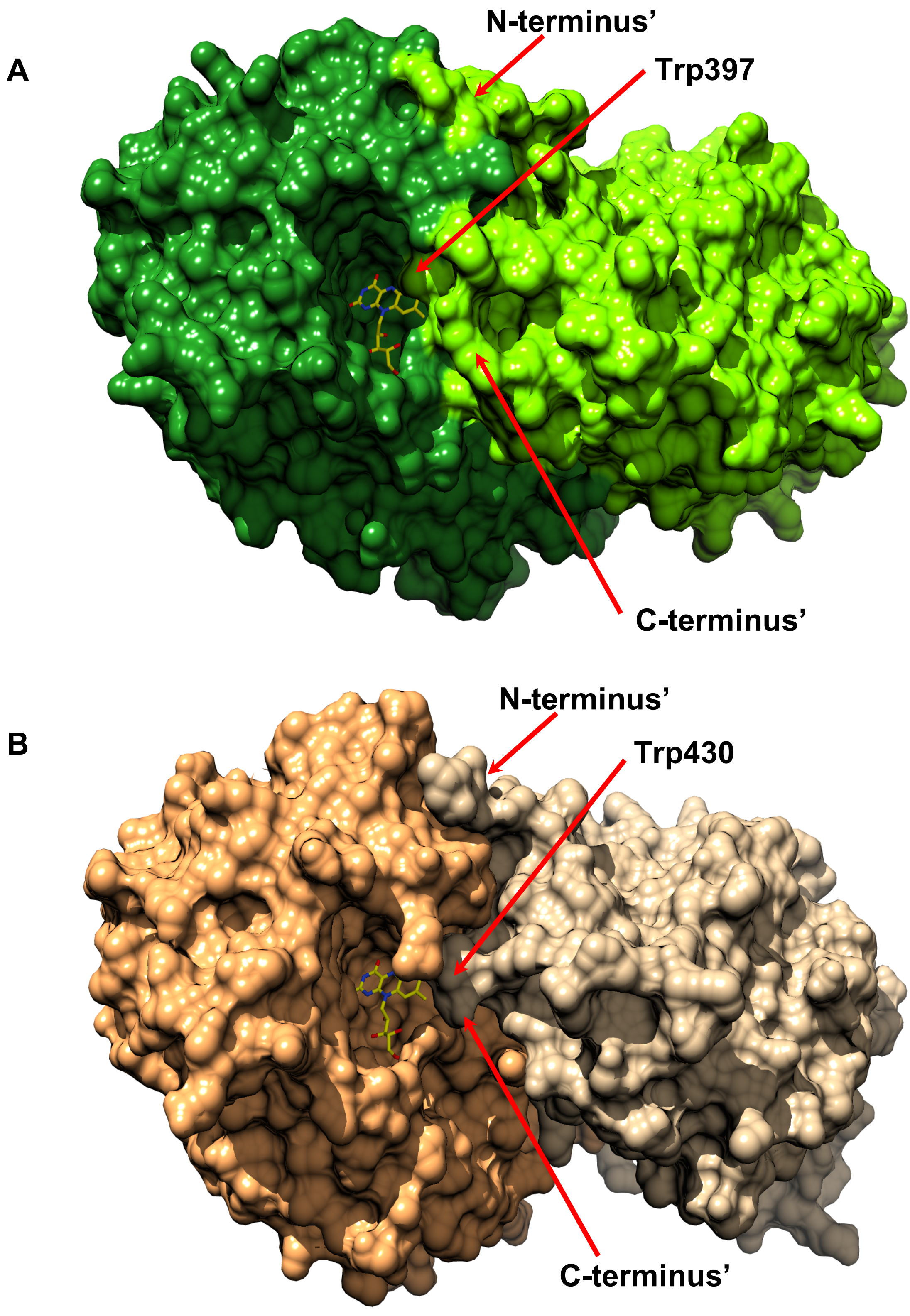

Figure S12. Comparison of *An*OYE8 dimer (forest green and light green) and *Bf*OYE4 dimer (sandy brown and tan) active site cavity architecture. FMN cofactor bound in the active site is shown with C atoms in yellow.

Front side

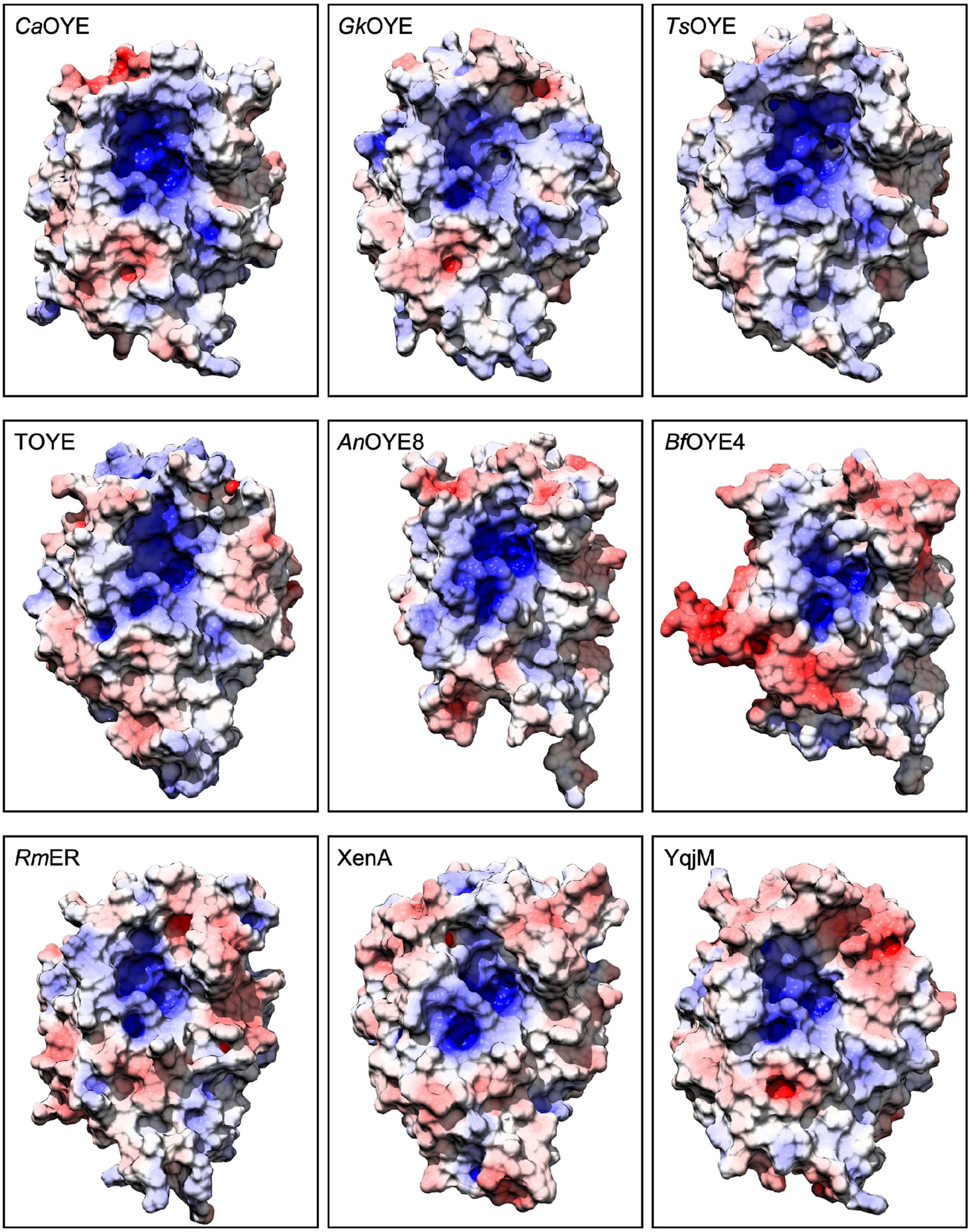

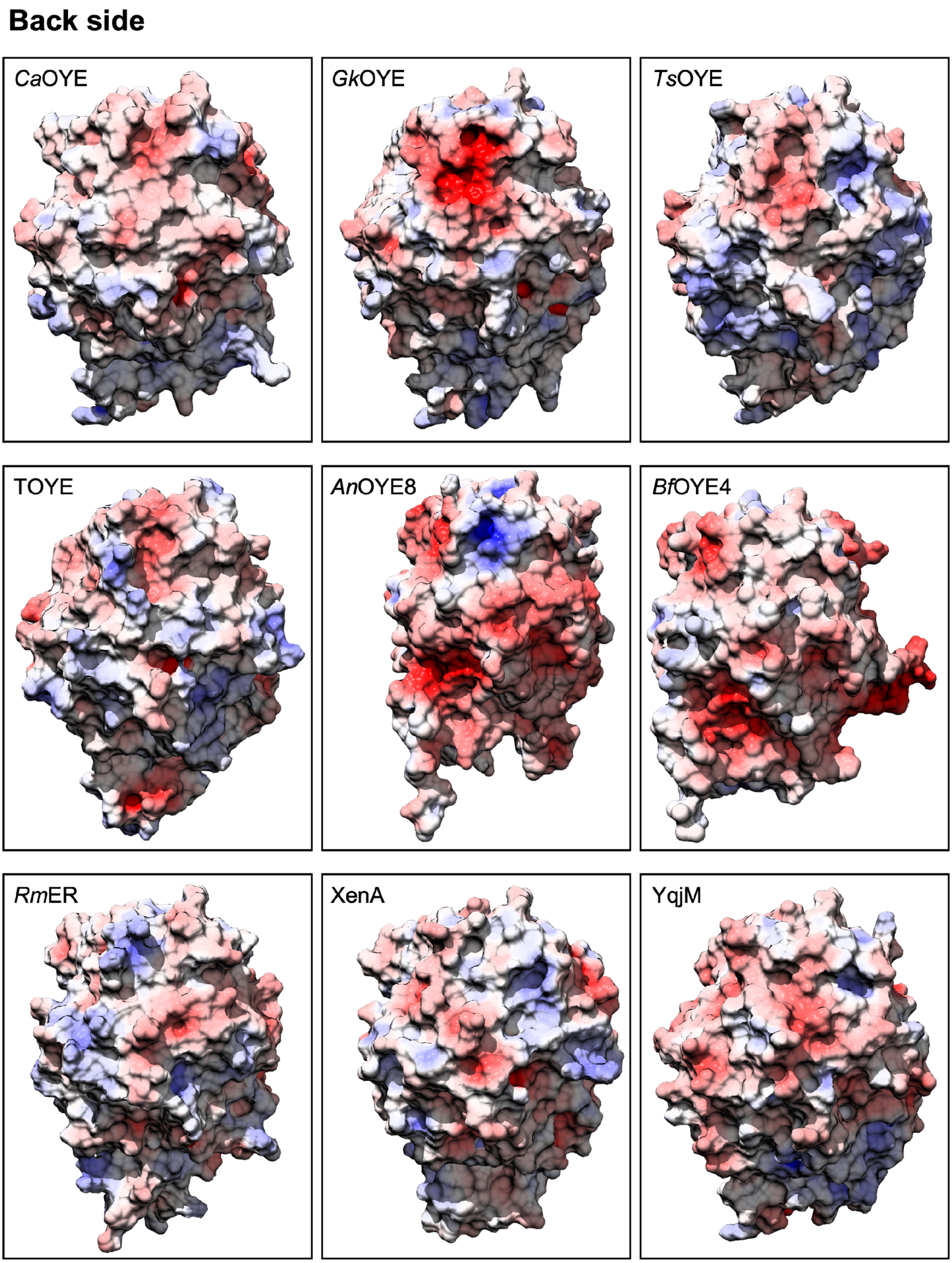

Figure S13. Comparative analysis of surface charge distribution among members of Class III OYEs, calculated at 150 mM NaCl, pH 8.0 and 310 K. Front side and back side. Density of negative potential is red, positive is blue and neutral is white. *Ca*OYE (pdb 7O0T); *Gk*OYE (pdb 3gr7); *Ts*OYE (pdb 3hf3); TOYE (pdb 3kru); *An*OYE8 (pdb 7QFX); *Bf*OYE4 (pdb 7BLF); *Rm*ER (pdb 5ocs); XenA (pdb 3L5L); YqjM (pdb 1z41).

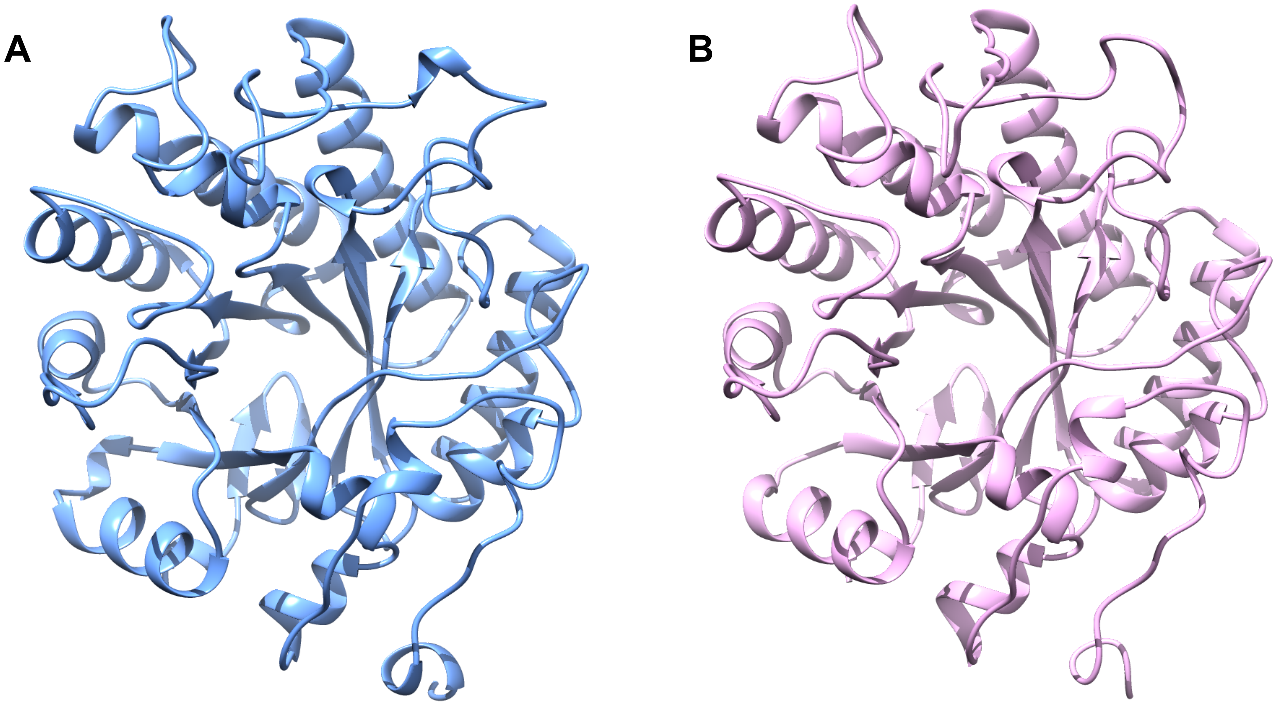

Figure S14. Models of *An*OYE2 (cornflower blue) and *Bf*OYE1 (pink) built using EasA structure as template. Both models share a similar architecture with a b-barrel surrounded by eight a-helices.

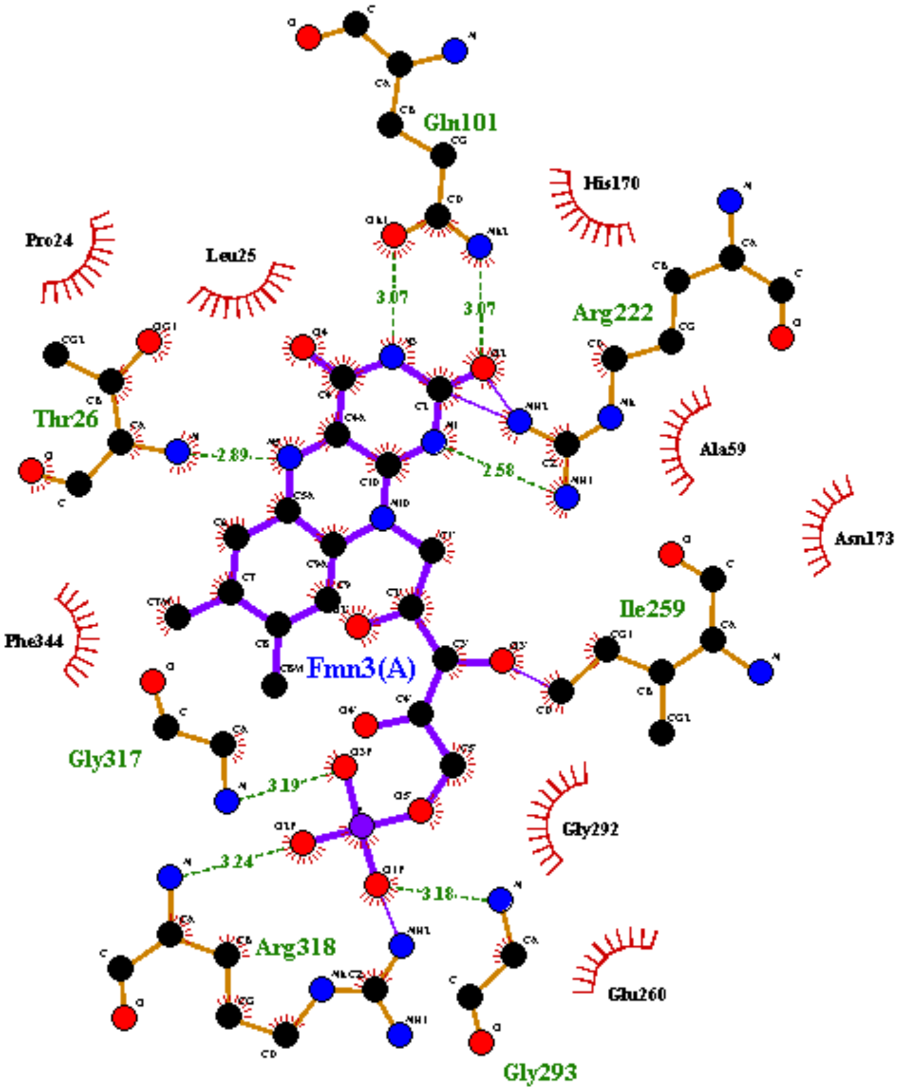

Figure S15. Schematic illustration of the interaction of FMN with active site residues of *An*OYE2. The thin green dotted lines illustrate hydrogen bonds.

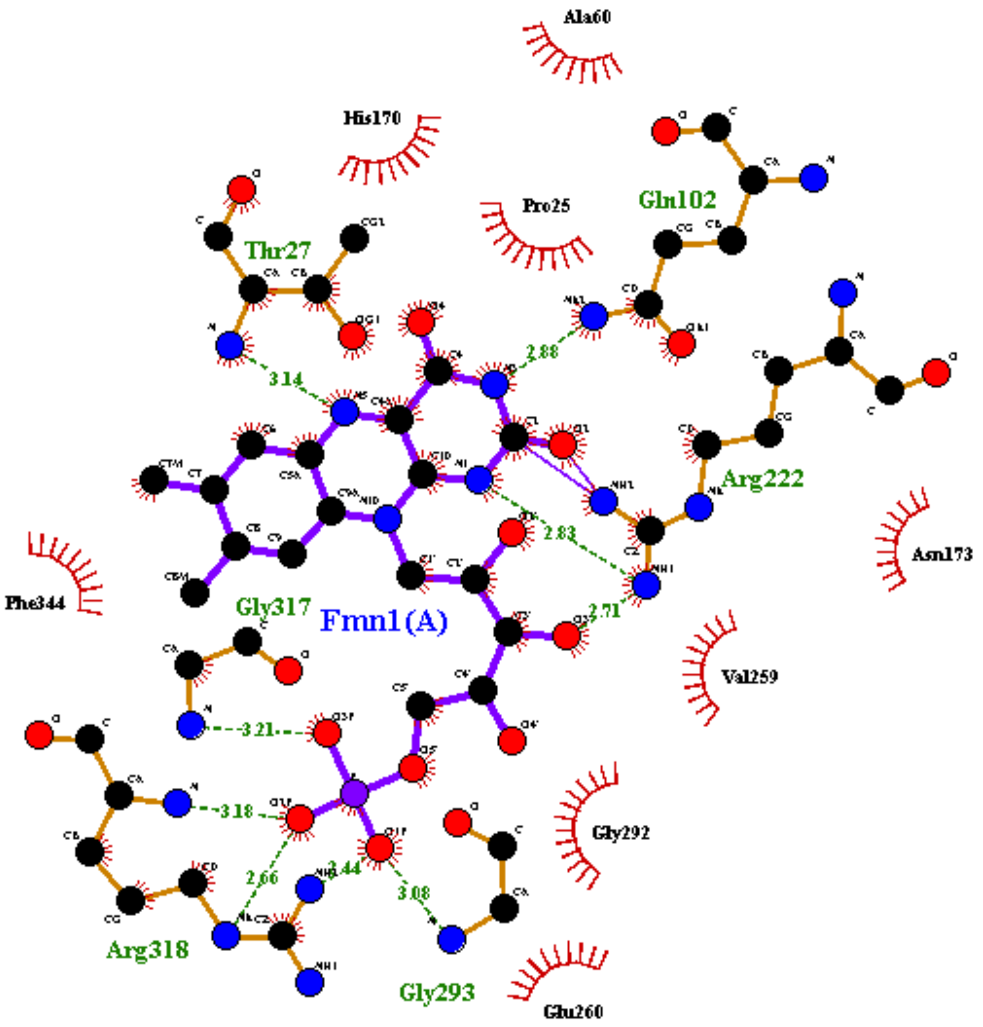

Figure S16. Schematic illustration of the interaction of FMN with active site residues of *Bf*OYE1. The thin green dotted lines illustrate hydrogen bonds.

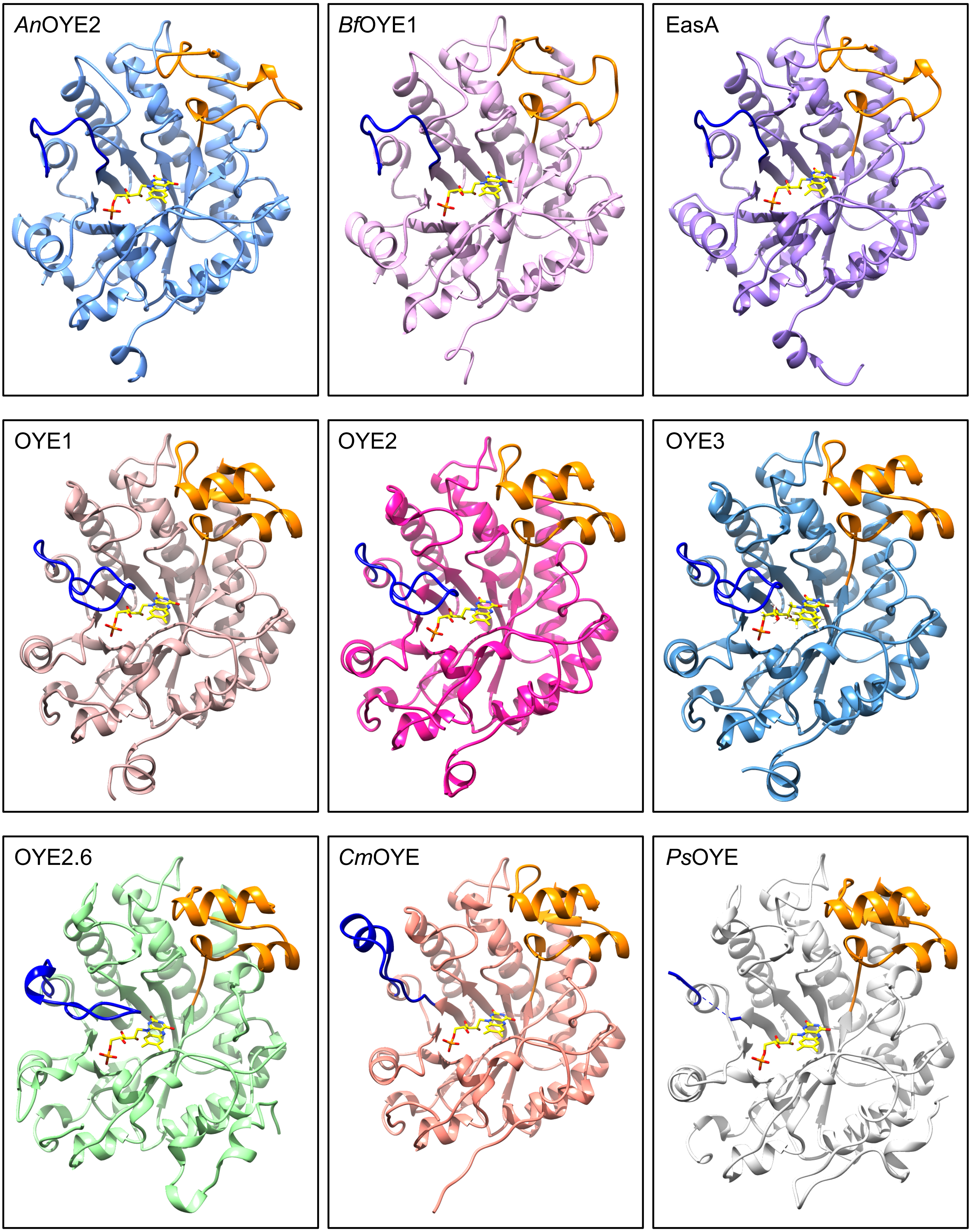

Figure S17. Comparison between loop 3 (orange) and loop 6 (blue) between *An*OYE2 and *Bf*OYE1 models and among Class II homologues crystal structures. EasA (4qnw), OYE1 (1oya), OYE2 (7BN7), OYE3 (3V4V), OYE2.6 (3TJL), *Cm*OYE (4TMB), *Ps*OYE (6AGZ).

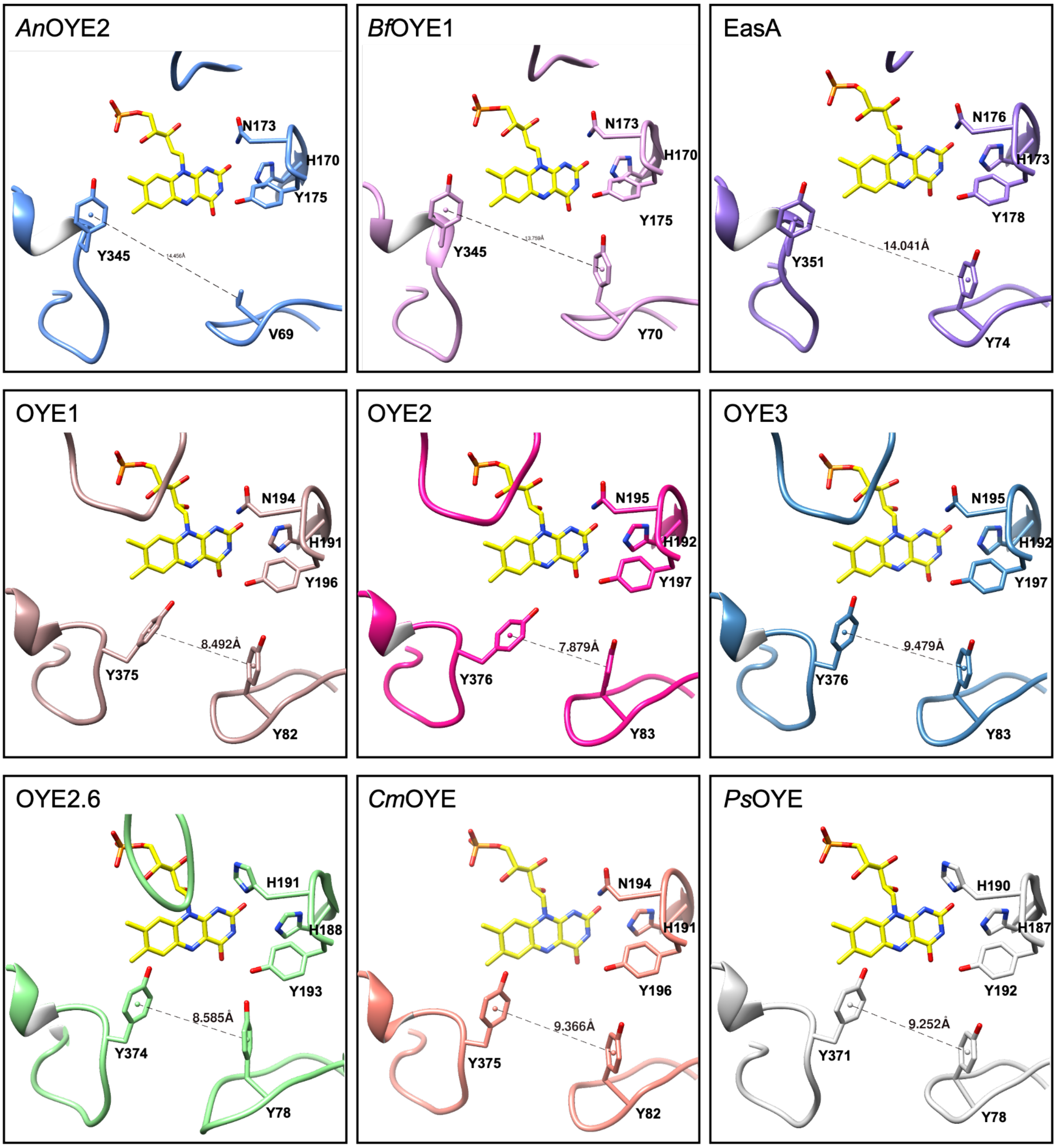

Figure S18. Pseudo-atom distances between the 'bounding' residues in Class II homologues. EasA (4qnw), OYE1 (1oya), OYE2 (7BN7), OYE3 (3V4V), OYE2.6 (3TJL), *Cm*OYE (4TMB), *Ps*OYE (6AGZ). The distances were measured with Chimera software.

Table S1. List of putative OYEs translated from the genomes of *Aspergillus niger* and *Botryotinia fukeliana* along with their corresponding nomenclature, protein sequence ID, Class, protein length, sub-cellular localization (modified from Nizam et al.)[3] and isoelectric point calculated by ProtParam (Expasy).

OYEs	Sequence ID	OYE Class	Sequence length	Molecular weight (kDa)	Sub-cellular localization	Isoelectric point
*An*OYE1	XP_001394816.2	II	533	58.69	Cytoplasm	5.61
***An*OYE2**	XP_001393044.1	II	369	41.15	Cytoplasm	6.02
*An*OYE3	XP_001390054.1	II	384	41.45	Mitochondria	5.83
*An*OYE4	XP_001401198.1	II	421	47.21	Cytoplasm	5.51
*An*OYE5	XP_001389993.1	III	420	45.06	Cytoplasm	6.32
*An*OYE6	XP_001389551.2	III	419	45.89	Cytoplasm	6.28
*An*OYE7	CAK48758.1	III	415	44.99	Cytoplasm	6.24
***An*OYE8**	XP_001399273.1	III	421	45.75	Cytoplasm	6.68
*An*OYE9	XP_001397404.1	III	443	47.42	Cytoplasm	6.13
*An*OYE10	XP_001391174.1	III	422	45.54	Cytoplasm	6.13
*An*OYE11	XP_001390672.2	V	412	44.82	Mitochondria	6.52
*An*OYE12	XP_001395504.2	V	418	45.81	Mitochondria	7.11
***Bf*OYE1**	XP_001558622.1	II	373	41.38	Cytoplasm	5.48
*Bf*OYE2	XP_001556041.2	II	370	41.26	Cytoplasm	5.42
***Bf*OYE4**	XP_001554780.1	III	439	47.72	Mitochondria	6.36
*Bf*OYE6	XP_001547575.1	V	443	49.11	Cytoplasm	6.01

Table S2. List of OYE sequences used for the phylogenetic analysis, and their accession numbers from the National Center for Biotechnology Information (NCBI), JGI database ([#]) or Phytozome (*).

Protein name	Organism	Accession number
AcaryoER1	*Acaryochloris marina* MBIC11017	ABW29811
AcaryoER3	*Acaryochloris marina* MBIC11017	ABW32756
AnabaenaER3	*Trichormus variabilis* ATCC 29413	ABA25236
*Ar*OYE1	*Ascochyta rabiei*	AHL17019
*Ar*OYE2	*Ascochyta rabiei*	AHL17020
*Ar*OYE3	*Ascochyta rabiei*	AHL17021
*Ar*OYE4	*Ascochyta rabiei*	AHL17022
*Ar*OYE5	*Ascochyta rabiei*	AHL17023
*Ar*OYE6	*Ascochyta rabiei*	AHL17024
*At*OPR1	*Arabidopsis thaliana*	NP_177794
*At*OPR2	*Arabidopsis thaliana*	NP_177795
*At*OPR3	*Arabidopsis thaliana*	NP_001077884
*Ca*OYE	*Chloroflexus aggregans*	WP_015941499
Chr-OYE1	*Cryseobacterium* sp. CA49	ALE60336
Chr-OYE2	*Cryseobacterium* sp. CA49	ALE60337
Chr-OYE3	*Cryseobacterium* sp. CA49	AHV90721
ChrR	Streptomyces sp. M7	RDS65860.1
ClER	*Clavispora lusitaniae* ATCC 42720	EEQ40235
*Cm*OYE	*Candida macedonensis* AKU4588	AB126227
*Cr*OYE1	*Chlamydomonas reinhardtii*	Cre01.g050150*
*Cr*OYE2	*Chlamydomonas reinhardtii*	Cre03.g210513*
*Cr*OYE3	*Chlamydomonas reinhardtii*	Cre17.g727300*
*Ct*OYE	*Chroococcidiopsis thermalis*	WP_015152687
CyanothER1	*Rippkaea orientalis* PCC 8801	ACK64210
CyanothER2	*Rippkaea orientalis* PCC 8801	ACK65723
CYE	*Kluyveromyces marxianus*	BAD24850
*Dr*ER	*Deinococcus radiodurans* R1	AAF11740
*Ea*ER2	*Ensifer adhaerens*	WP_090295514
EBP1	*Candida albicans*	AAA18013
FgaOx3 (EasA)	*Aspergillus fumigatus*	XP_756133
FgaOx3$_{Pc}$	*Penicillum comune*	AFM84626.1
FgaOx3$_{Pr}$	*Penicillum roqueforti*	CDM33403.1
FOYE-1	*Ferrovum* sp. JA12	KRH78075
*Geo*ER	*Geobacillus* sp. #30	BAO37313
*Gk*OYE	*Geobacillus kaustophilus* HTA426	BAD76617
GloeoER	*Gloeobacter violaceus* PCC 7421	BAC91769
GluER	*Gluconobacter oxydans* 621H	AAW60280
*Gs*OYE	*Galdieria sulphuraria*	XP_005703492
HYE1	*Ogataea angusta*	AAN09952
HYE2	*Ogataea angusta*	AAN09953
KYE1	*Kluyveromyces lactis*	AAA98815
LacER	*Lactobacillus paracasei*	ADK19581
*Le*OPR1	*Solanum lycopersicum*	NP_001234781
*Le*OPR2	*Solanum lycopersicum*	NP_001233868
*Le*OPR3	*Solanum lycopersicum*	NP_001233873
LyngbyaER1	*Lyngbya* sp. PCC 8106	EAW37813
*Mc*OYE1	*Mucor circinelloides* CBS 277.49	2762605897[#]
*Mc*OYE2	*Mucor circinelloides* CBS 277.49	2762605847[#]
*Mc*OYE3	*Mucor circinelloides* CBS 277.49	2762608038[#]
*Mc*OYE4	*Mucor circinelloides* CBS 277.49	2762608163[#]
*Mc*OYE5	*Mucor circinelloides* CBS 277.49	2762608555[#]
*Mc*OYE6	*Mucor circinelloides* CBS 277.49	2762609952[#]
*Mc*OYE7	*Mucor circinelloides* CBS 277.49	2762610390[#]
*Mc*OYE8	*Mucor circinelloides* CBS 277.49	2762604297[#]
*Mc*OYE9	*Mucor circinelloides* CBS 277.49	2762605021[#]
*Mc*OYE10	*Mucor circinelloides* CBS 277.49	2762607661[#]

*Mg*ER	*Meyerozyma guilliermondii* ATCC 6260	EDK41665
MR	*Pseudomonas putida*	AAC43569
NCR	*Zymomonas mobilis*	AAV90509
NemA	*Escherichia coli*	BAA13186
NerA	*Agrobacterium tumefaciens*	CAA74280
NospuncER1	*Nostoc punctiforme* PCC 73102	ACC84535
NostocER1	*Nostoc* sp. PCC 7120	BAB73564
OYE1	*Saccharomyces pastorianus*	Q02899
OYE2	*Saccharomyces cerevisiae* S288C	Q03558
OYE2.6	*Scheffersomyces stipitis* CBS 6054	ABN66026
OYE2p	*Saccharomyces cerevisiae* YJM1341	AJV32222.1
OYE3	*Saccharomyces cerevisiae* S288C	P41816
OYE*Ro*2	*Rhodococcus opacus* 1CP	ALL54975
PETNR	*Enterobacter cloacae*	AAB38683
*Pf*ER2	*Pseudomonas fluorescens*	WP_038581871
Pfvc	*Arthrobacter* sp. JBH1	AFF18622
PpoER1	*Paenibacillus polymyxa* CR1	AHC19521
PpoER2	*Paenibacillus polymyxa* CR1	AIW41616
PpoER3	*Paenibacillus polymyxa*	QBR53093
*Ps*OYE	*Pichia* sp.AKU4542 (formerly, *Toluropsis* sp.)	6AGZ_A
*Pv*ER1	*Pseudomonas veronil*	WP_057005725
*Pv*ER2	*Pseudomonas veronil*	WP_024074791
*Rhr*ER2718	*Rhodococcus rhodochrous* ATCC 17895	AMD82542
*Rm*ER	*Cupriavidus metallidurans* CH34	ABF11721
SYE1	*Shewanella oneidensis* MR-1	AAN55488
SYE3	*Shewanella oneidensis* MR-1	AAN57126
SYE4	*Shewanella oneidensis* MR-1	AAN56390
SynER	*Synechococcus elongatus* PCC 7942	ABB56505
TOYE	*Thermoanaerobacter pseudethanolicus* ATCC	ABY93685
*Ts*OYE	*Thermus scotoductus* SA-01	CAP16804
XenA	*Pseudomonas putida*	AAF02538
XenB	*Pseudomonas fluorescens*	AAF02539
YersER	*Yersinia bercovieri*	WP_032896199
YqiG	*Bacillus subtilis*	QBR53092
YqjM	*Bacillus subtilis*	BAA12619

The protein sequences of pQR1439, pQR1440, pQR1442, pQR1443, pQR1445, pQR1446, pQR1907, pQR1908, pQR1909 are from Dobrijevic et al.[4]

Table S3. Comparison of *An*OYE8 and *Bf*OYE4 characteristics with those of other Class III OYEs. Parameters referring to primary sequence have been calculated with Protparam Expasy; oligomerization states in solution have been obtained from literature data and derived from size exclusion, native gel or light scattering measurements; in crystal oligomerization data were deduced from crystallographic structures, as well as interface area and number of interactions (Hydrogen bonds, Hb, and Salt bridges, Sb) involving the dimerization surface, calculated by PISA software; T_{opt} corresponds to the optimal temperature for enzymatic activity and it has been deduced from literature data, while T_m corresponds to melting temperature of the corresponding recombinant enzymes, measured either by Fluorescence (ThermoFAD or Thermofluor) or Circular dichroism.

					**Oligomerization**						
	**Enzyme**	**Sequence length (aa)**	**Arg/Lys ratio**	**Total proline content (%)**	**In solution**	**In crystal**	**Interface (Å^2, %tot)**	**Interactions (Hb, Sb)**	**T_{opt} (°C)**	**T_m (°C)**	**References**
Thermostable OYEs	*Ca*OYE	354	7.0	8.2	monomer	dimer	1451 (10.4%)	(16; 8)	-	79	5
	FOYE1	354	1.6	6.2	-	-	-	-	50 ($t_{1/2}$= 5 h)	-	6
	*Gk*OYE	340	2.2	6.2	tetramer	dimer	1211 (8.9%)	(16; 0)	70	76-82	7
	GeoER	340	1.7	5.0	trimer or tetramer	-	-	-	70	-	8
	*Ts*OYE	349	4.6	8.0	dimer	octamer of dimers	1273 (9.3%)	(16; 2)	65	-	9 a,b
	TOYE	337	0.7	3.6	tetramer, octamer, dodecamer	dimer of dimers	1073 (8.0%)	(15; 0)	-	> 70	10
Not-thermostable OYEs	*An*OYE8	421	1.1	6.4	dimer	dimer	2807	(18; 8)	-	44	This work
	*Bf*OYE4	439	1.3	7.1	monomer; dimer; higher oligomeric states	dimer	2181	(14; 2)	-	43	This work
	YqjM	338	0.9	3.8	tetramer	dimer of dimers	1152 (8.5%)	(13; 0)	25-30 ($t_{1/2}$= 9 days)	-	11 a,b
	XenA	363	2.9	6.1	dimer	dimer	1346 (9.7%)	(2; 2)	-	50.4	12 a,b

*Rm*ER	371	2.0	7.0	monomer	dimer	2132 (14%)	(28; 7)	35	-	13 a,b
*Dr*ER	370	4.4	6.2	dimer	-	-	-	30	-	13a
*Chr*OYE3	350	1.3	4.0	dimers, tetramers	-	-	-	35 ($t_{1/2}$= 233.5 h)	-	14
*Rhr*ER2718	367	3.4	6.3	dimer	-	-	-	40	-	15
OYERo2	366	6.3	6.8	dimer/ tetramer conc. dependent	-	-	-	up to 20	-	16
PfvC	365	12.5	6.0	-	-	-	-	-	-	17
*Cr*OYE1	410	2.5	9.0	-	-	-	-	40 ($t_{1/2}$= 5.7 days)	-	18
AnabaenaER3	357	4.8	7.0	-	-	-	-	-	-	19
GloeoER	354	10.7	6.2	-	-	-	-	-	-	19

Table S~~3~~4. X-ray crystallographic data collection and refinement statistics for *An*OYE8.

	***An*OYE8 (pdb 7QFX)**
Wavelength	0.87
Resolution range	39.95-2.80 (2.91-2.80)
Space group	C121
Unit cell	157.533 65.388 181.417<br>90 107.756 90
Total reflections	208496 (18267)
Unique reflections	43316 (4327)
Multiplicity	4.8 (4.2)
Completeness (%)	99 (95)
Mean I/sigma(I)	13.2 (3)
Wilson B-factor	47.8
R-merged	0.131 (0.595)
R-work	0.241
R-free	0.260
Number of non-hydrogen atoms	12834
macromolecules	12596
ligands	144
solvent	83
Protein residues	1639
RMS (bonds)	0.012
RMS (angles)	1.63
Ramachandran favored (%)	91.0
Ramachandran allowed (%)	9.0
Ramachandran outliers (%)	0.0
Rotamer outliers (%)	17.39
Average B-factor	22.83
macromolecules	22.47
ligands	43.49
solvent	41.59

Table S45. Quality of *An*OYE2 and *Bf*OYE1 models.

**Protein**	***An*OYE2**	***Bf*OYE1**
Identity with template (EasA, pdb 4qnw) (%)	51.93	52.91
Coverage (%)	98.91	97.58
GQME	0.85	0.84
QMEAN6	-0.55	-0.93
QMEAN_DisCo_Global	0.82	0.81
MOLPROBITY_SCORE	1.7	1.57
Ramachandran favored (%)	94.18	93.91

Table S~~5~~6. Oligonucleotides used for PCR amplification of new ER sequences and cloning.
Restriction sites are shown in bold. Modifications introduced to mutate the original sequences are underlined.

	sequence (5′ → 3′)	Purpose
AnOYE2.1_for	GGCGTGGAGGTGGTGTTGGAGACGC	
AnOYE 2.2_rev	CCACATTCCACGAGTACCGCCTCGAGCGC	
AnOYE 2.3_for	CGCCGGTC**CATATG**TGCTCC	*Nde*I site introduction
AnOYE 2.4_rev	GCGCTTGTAT**GGATCC**AATATCAGC	*Bam*HI site introduction
AnOYE 8.1_for	CGTTCGAGACTAGGAGGTGCCGCGTGAG	
AnOYE 8.2_rev	CGCCCTGAGTTTTGCCGCTTAGACGGAGG	
AnOYE 8.3_for	GAACCGTCTCGGGGTATCCCCAATGTGCCAG	Removal of intronic sequence
AnOYE 8.4_rev	CTGGCACATTGGGGATACCCCGAGACGGTTC	Removal of intronic sequence
AnOYE 8.5_for	GAAGAGCACA**CATATG**AAGGACATCAAGG	*Nde*I site introduction
AnOYE 8.6_rev	CCCGTCAAGTCTA**AAGCTT**CGTACATG	*Hind*III site introduction
BfOYE1.1_for	ATTCCGCCGAACACCCCACT	
BfOYE 1.2_rev	CTCCCAGGCGCAAAGGCTCT	
BfOYE 1.3_for	TACTAAACA**CATATG**TCTCCATCCACAC	*Nde*I site introduction
BfOYE 1.4_rev	GGTATCTATCTAT**GGATCC**ATGCGTC	*Bam*HI site introduction
BfOYE 1.5_for	CTGGAATGGTGTCAGCCCAATTCTC	Putative glycosylation site suppression (T285V)
BfOYE 1.6_rev	GAGAATTGGGCTGACACCATTCCAG	Putative glycosylation site suppression (T285V)

Table S67. Optimized conditions for *An*OYE2, *An*OYE8 and *Bf*OYE1 recombinant production: expression host, temperature, riboflavin concentration and final yield.

**Enzyme**	**Expression host strain**	**Temperature (°C)**	**[Riboflavin] (μM)**	**Yield (mg/L)**
*An*OYE2	*E. coli* BL21 (DE3) Arctic®	12	25	13
	E. coli BL21 (DE3)	16	100	18
*An*OYE8	*E. coli* BL21 (DE3) Arctic®	12	25	9
	E. coli BL21 (DE3)	16	100	10
*Bf*OYE1	*E. coli* BL21 (DE3)	25	25	42

References

1. Oberdorfer, G.; Steinkellner, G.; Stueckler, C.; Faber, K.; Gruber, K. Stereopreferences of old yellow enzymes: Structure correlations and sequence patterns in enoate reductases. *ChemCatChem* **2011**, *3*, 1562–1566.
2. Robescu, M.S.; Cendron, L.; Bacchin,A.; Wagner, K.; Reiter, T.C.; Janicki, I.; Merusic, K.; Illek, M.; Bergantino, R.; Hall, M. Asymmetric Proton Transfer Catalysis by Stereocomplementary Old Yellow Enzymes for C=C-Bond Isomerization Reaction. *under revision*
3. Nizam, S.; Verma, S.; Borah, N.N.; Gazara, R.K.; Verma, P.K. Comprehensive genome-wide analysis reveals different classes of enigmatic old yellow enzyme in fungi. *Sci. Rep.* **2014**, *4*, 4013–4024.
4. Dobrijevic, D.; Benhamou, L.; Aliev, A.E.; Mendez-Sanchez, D.; Dawson, N.; Baud, D.; Tappertzhofen, N.; Moody, T.S.; Orengo, C.A.; Hailes, H.C.; Ward, J.M. Metagenomic ene-reductases for the bioreduction of sterically challenging enones. *RSC Adv.* **2019**, *9*, 36608–36614.
5. Robescu, M.S.; Niero, M.; Loprete, G.; Cendron, L.; Bergantino, E. A new thermophilic end-reductase from the filamentous anoxygenic phototrophic bacterium *Chloroflexus aggregans*. *Microorganisms* **2021**, 9, 953.
6. Scholtissek, A.; Ullrich, S.R.; Mühling, M.; Schlömann, M.; Paul, C.E.; Tischler, D. A thermophilic-like ene-reductase originating from an acidophilic iron oxidizer. *Appl. Microbiol. Biotechnol.* **2017**, *101*, 609-619.
7. Schittmayer, M.; Glieder, A.; Uhl, M.K.; Winkler, A.; Zach, S.; Schrittwieser, J.H.; Kroutil, W.; Macheroux, P.; Gruber, K.; Kambourakis, S.; Rozzell, J.D.; Winkler, M. Old yellow enzyme-catalyzed dehydrogenation of saturated ketones. *Adv. Synth. Catal.* **2011**, *353*, 268-274.
8. Tsuji, N.; Honda, K.; Wada, M.; Okano, K.; Ohtake, H. Isolation and characterization of a thermotolerant ene-reductase from *Geobacillus* sp. 30 and its heterologous expression in *Rhodococcus opacus*. *Appl. Microbiol. Biotechnol.* **2014**, *98*, 5925–5935.
9. a) Opperman, D.J.; Piater, L.A.; van Heerden, E. A novel chromate reductase from *Thermus scotoductus* SA-01 related to Old Yellow Enzyme. *J. Bacteriol.* **2008**, *190*, 3076–3082. b) Opperman, D.J.; Sewell, B.T.; Litthauer, D.; Isupov, M.N.; Littlechild, J.A.; van Heerden, E. Crystal structure of a thermostable Old Yellow Enzyme from *Thermus scotoductus* SA-01. *Biochem. Biophys. Res. Comm.* **2010**, *393*, 426-431.
10. Adalbjornsson, B.V.; Toogood, H.S.; Fryszkowska, A.; Pudney, C.R.; Jowitt, T.A.; Leys, D.; Scrutton, N.S. Biocatalysis with thermostable enzymes: structure and properties of a thermophilic ene-reductase related to Old Yellow Enzyme. *ChemBioChem* **2010**, *11*, 197–207.
11. a) Kitzing, K.; Fitzpatrick, T.B.; Wilken, C.; Sawa, J.; Bourenkov, G.P.; Macheroux, P.; Clausen, T. The 1.3 A° crystal structure of the flavoprotein YqjM reveals a novel class of old yellow enzymes. *J. Biol. Chem.* **2005**, *280*, 27904-27913. b) Pesic, M.; Fernandez-Fueyo, E.; Hollmann, F. Characterization of the Old Yellow Enzyme homolog from *Bacillus* subtilis (YqjM). *ChemistrySelect* **2017**, *2*, 3866-3871.
12. a) Griese, J.J.; Jakob, R.P.; Schwarzinger, S.; Dobbek, H. Xenobiotic reductase A in the degradation of quinoline by *Pseudomonas putida* 86: physiological function, structure and mechanism of 8-hydroxycoumarin reduction. *J. Mol. Biol.* **2006**, *361*, 140-152. b) Yanto, Y.; Yu, H.-H.; Hall, M.; Bommarius, A.S. Characterization of xenobiotic reductase A (XenA): study of active site residues, substrate spectrum and stability. *Chem. Commun.* **2010**, *46*, 8809-8811.
13. a) Litthauer, S.; Gargiulo, S.; van Heerden, E.; Hollmann, F.; Opperman, D.J. Heterologous expression and characterization of the ene-reductases from *Deinococcus radiodurans* and *Ralstonia metallidurans*. *J. Mol. Catal. B Enzym.* **2014**, *99*, 89–95. b) Opperman, D.J. Structural investigation into the C-terminal extension of the ene-reductase from *Ralstonia (Cupriavidus) metallidurans. Proteins* **2017**, *85*, 2252–2257.
14. Xu, M.-Y.; Pei, X.-Q.; Wu, Z.-L. Identification and characterization of a novel "thermophilic-like" Old Yellow Enzyme from the genome of *Chryseobacterium* sp. CA49. *J. Mol. Catal. B Enzym.* **2014**, *108*, 64–71.
15. Chen, B.-S.; Médici, R.; van der Helm, M.P.; van Zwet, Y.; Gjonaj, L.; van der Geest, R.; Otten, L.G.; Hanefeld, U. *Rhodococcus* strains as source for ene-reductase activity. *Appl. Microbiol. Biotechnol.* **2018**, *102*, 5545-5556.
16. Riedel, A.; Tischler, D. Functional characterization and stability improvement of a 'thermophilic-like' ene-reductase from *Rhodococcus opacus* 1CP. *Front. Microbiol.* **2015**, *6*, 1073.
17. . Husserl, J.; Huges, J.B.; Spain, J.C. Key enzymes enabling the growth of *Arthrobacter* sp. Strain JBH1 with nitroglycerin as the sole source of carbon and nitrogen. *Appl. Environ. Microbiol.* **2012**, *78*, 3649-3655.
18. Böhmer, S.; Marx, C.; Gomez-Baraibar, A.; Nowaczyk, M.M.; Tischler, D.; Hemschemeier, A.; Happe, T. Evolutionary diverse *Chlamydomonas reinhardtii* Old Yellow Enzymes reveal distinctive catalytic properties and potential for whole-cell biotransformations. *Algal Res.* **2020**, *50*, 101970.
19. Fu, Y.; Castiglione, K.; Weuster-Botz, D. Comparative characterization of novel ene-reductases from Cyanobacteria. *Biotechnol. Bioeng.* **2013**, *110*, 1293–1301.
